# Polyoxometalate@Metal–Organic Framework‐Based Imprinted Electrochemical Sensor for Dopamine Detection

**DOI:** 10.1002/smsc.70380

**Published:** 2026-08-21

**Authors:** Wenjing Wang, Di Wang, Srivatsan K. Vasantham, Hongrong Hu, Yan Liu, Andreas H. Schäfer, George Mathew, Navid Hussain, Masooma Ibrahim, Jasmin Aghassi‐Hagmann, Annie K. Powell, Michael Hirtz

**Affiliations:** ^1^ Institute of Nanotechnology (INT) Karlsruhe Institute of Technology (KIT) Karlsruhe Germany; ^2^ Karlsruhe Nano Micro Facility (KNMFi) Karlsruhe Institute of Technology (KIT) Karlsruhe Germany; ^3^ nanoAnalytics GmbH Münster Germany; ^4^ Institute of Inorganic Chemistry (AOC) Karlsruhe Institute of Technology (KIT) Karlsruhe Germany; ^5^ Institute for Quantum Materials and Technologies (IQMT) Karlsruhe Institute of Technology (KIT) Karlsruhe Germany

**Keywords:** dopamine, electrochemical sensor, metal–organic frameworks, molecularly imprinted polymers, polyoxometalates

## Abstract

Electrochemical determination of dopamine (DA) has received considerable attention in recent years, with current research primarily focused on improving sensor sensitivity and selectivity. In this study, a printed electrochemical sensor modified with a hybrid material comprising polyoxometalates (POMs), metal–organic frameworks (MOFs), and molecularly imprinted polymers (MIPs) is fabricated for DA detection. The POM@MOF component enhances charge transfer, thereby improving sensitivity, while the MIP acts as a selective receptor for DA, providing high selectivity. The fabricated sensor exhibits selective recognition of DA from interferents with similar electrochemical behaviors using cyclic voltammetry (CV), and the CV responses are linear with DA concentration in the range 200–800 μM, with a limit of detection (LOD) of 59.03 μM. Moreover, the sensors show good stability, regeneration ability, and reproducibility. The results demonstrate that the proposed material can facilitate the electrochemical sensing performance toward DA, providing a basis for the future design and optimization of material‐based electrochemical DA sensing platforms.

## Graphical abstract

A synergistic assembly of molecularly imprinted polymer (MIP) and polyoxometalates@metal–organic framework (POM@MOF) is utilized to modify the electrode surface of a dopamine electrochemical sensor. POM@MOF enhances the sensitivity, while MIP enables selective discrimination against interferents. The resulting sensor exhibits good dopamine sensing performance in solution with high stability and regeneration ability.

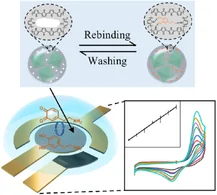

## 1 Introduction

Dopamine (DA), an essential catecholamine neurotransmitter widely distributed in humans and some mammals [1, 2], plays critical roles in learning [3], cognition [4], and the regulation of gastrointestinal motility [5] and blood pressure [6]. Abnormal DA levels in the body have been proven to be closely associated with neurological disorders such as Parkinson's disease [7], Alzheimer's disease [8], motor and urinary dysfunction [9], and depression [10]. Moreover, the effects of DA on immune cells have recently attracted growing scientific interest [11]. Therefore, the qualitative and quantitative detection of DA in vivo or in vitro is crucial for monitoring physiological conditions and facilitating the early clinical diagnosis of associated diseases. Numerous traditional analytical methods with high sensitivity and selectivity have been developed for the determination of DA, including high‐performance liquid chromatography [12, 13], mass spectrometry [14, 15], and capillary electrophoresis [16, 17]. However, these laboratory‐based techniques are time‐consuming, require expensive and sophisticated equipment, and demand skilled operators for accurate readouts. Therefore, there is a strong demand for the development of DA detection techniques that are user‐friendly and capable of real‐time analysis.

Electrochemical sensors have gained significant attention as effective candidates for the precise detection of DA, due to their cost‐effectiveness, rapid response, and high sensitivity and selectivity [18, 19]. Additionally, their compatibility with miniaturization and integration into electronic systems further highlights their potential for point‐of‐care testing. In developing electrochemical methods for DA detection, two main factors, sensitivity and selectivity, must be considered, as DA typically has a low concentration in biological samples and coexists with several interfering species with overlapping electrochemical signals, complicating the detection of DA. To address the limitations mentioned above, much effort has been made to modify the sensors with functional materials, such as metals and metal oxides [20, 21], conductive polymers [22], carbon‐based nanomaterials [23], and metal–organic frameworks (MOFs) [24], in order to enhance detection performance.

Polyoxometalates (POMs) are a family of metal–oxide clusters with tailorable structures and versatile physicochemical properties [25]. Owing to their capacity for reversible multi‐electron acceptance and transfer properties without structural changes, POMs can function as “electron sponges” with rapid redox kinetics, establishing them as promising electrode active materials for electrochemical sensors aimed at improving sensitivity [26]. However, the high solubility of POMs in water limits their direct application as modifiers or active components on electrochemical sensor surfaces, since they tend to leach into the solution during measurements, resulting in poor sensor stability. As a strategy to improve their operational stability in liquid environments, POMs have been anchored on various high‐surface‐area substrates or incorporated into functional materials, including porous silica [27], metal oxides [28], carbon‐based materials [29], polymers [30], and porous framework materials [31]. MOFs, a well‐known class of porous materials with a large surface area and the ability to absorb various guest molecules, can provide a possible support to immobilize the POMs on the surface [32]. Meanwhile, MOFs have demonstrated considerable potential in electrochemical sensors due to their tunable chemical functionalities, as exemplified by sulfonate‐functionalized MOF‐808 for modulating the selectivity and electrochemical reaction rates [33], MOF/carbon nanotubes composite for the dual fluorescence and electrochemical detection of DA [34], and 2D conductive MOFs enabling facet‐dependent control over DA–electrode interfacial interactions [35]. Therefore, the composites of POM and MOF have attracted interest in DA electrochemical sensing, and some related studies have been reported [36, 37, 38]. However, the selectivity of most POM@MOF‐based sensors primarily relies on the nonspecific affinity between DA and the composites, which is insufficient to discriminate structurally similar molecules in complex matrices. Molecularly imprinted polymers (MIPs) represent a well‐established strategy for achieving specific selectivity toward target analytes. MIPs are synthesized by polymerizing a crosslinked polymer matrix in the presence of the target molecules, which are then removed to generate specific recognition cavities within the polymer network that are complementary in shape, size, and chemical functionality to the target analytes [39]. MIPs are extensively utilized as recognition elements in electrochemical sensors due to their straightforward synthesis, cost‐effectiveness, precise selectivity, and high stability [40, 41, 42]. Nonetheless, the combination of POM@MOF and MIP in the field of electrochemical sensing remains insufficiently investigated.

Hence, in this work, a hybrid POM@MOF/MIP material was synthesized as an electrode modifier of the electrochemical sensor for DA detection. H_3_PW_12_O_40_ (PW_12_), a widely studied Keggin‐type POM, and MIL‐101(Cr), a water‐stable MOF, were employed to enhance sensitivity, while pyrrole was used in the synthesis of the polypyrrole (PPy) MIP to impart selectivity. Following synthesis, the PW_12_@MIL‐101(Cr)/PPy‐MIP was deposited on the electrode of the electrochemical sensor via capillary printing, which offers significant advantages in device miniaturization and direct on‐chip sensor fabrication over conventional spin coating or drop casting methods. Under conditions of a pyrrole‐to‐DA ratio of 4:1, deposition of two printed modifier layers on the electrode, and a pH 1.0 electrolyte, the prepared PW_12_@MIL‐101(Cr)/PPy‐MIP sensor achieved good sensitivity and selectivity toward the electrochemical detection of DA. Moreover, it maintained excellent stability, regeneration ability, and reproducibility in DA detection.

## 2 Results and Discussion

### 2.1 Design of Material and Sensor Architecture

**SCHEME 1:**
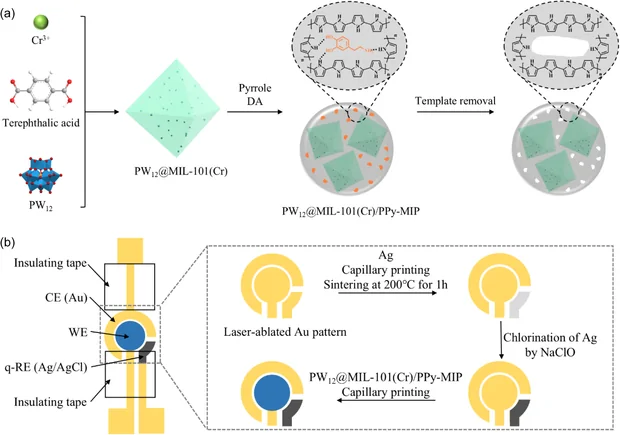
(a) Synthesis process of PW_12_@MIL‐101(Cr)/PPy‐MIP, (b) Configuration (left) and fabrication process (right) of the PW_12_@MIL‐101(Cr)/PPy‐MIP‐based sensor.

The synthesis strategy of PW_12_@MIL‐101(Cr)/PPy‐MIP is illustrated in Scheme 1a. PW_12_@MIL‐101(Cr), first prepared via a one‐pot method, was copolymerized with pyrrole and DA at a pyrrole‐to‐DA ratio of 4:1 (applied in all cases unless otherwise specified), producing a PPy matrix that entrapped both PW_12_@MIL‐101(Cr) and DA. Removing DA leaves behind specific binding cavities complementary to DA within the polymer network. The MOF MIL‐101(Cr) serves as a porous carrier that enables the dispersion of the incorporated POM, PW_12_, and simultaneously prevents its dissolution in the electrolyte solution. Moreover, the synergistic effect of π–π conjugation between MOFs and pyrrole and the ionic interactions between POMs and pyrrole facilitate pyrrole polymerization and stabilize PPy on the surface of the framework [43]. The pristine PW_12_@MIL‐101(Cr) appeared as a green powder. After polymerization with pyrrole, the resulting composite turned black (Figure S1), which is consistent with the characteristic color of PPy. The synthesized hybrid material was subsequently deposited on the electrode as a surface modifier, as illustrated in Scheme 1b.

DA is an electroactive molecule that undergoes reversible redox reactions at the electrode surfaces, where it is oxidized at specific potentials to form its corresponding quinone, which can subsequently be reduced back to DA (Figure S2). These redox processes can generate measurable current responses, enabling electrochemical detection [44]. In this study, a three‐electrode system comprising a working electrode (WE), counter electrode (CE), and reference electrode (RE) was employed to investigate the electrochemical behavior of DA at the surface of WE. Scheme 1b illustrates the schematic configuration and fabrication process of the PW_12_@MIL‐101(Cr)/PPy‐MIP electrochemical sensor. In this design, gold served as the base material for all the electrodes, which were thereafter modified according to their functional requirements. The WE was functionalized with PW_12_@MIL‐101(Cr)/PPy‐MIP to enhance both sensitivity and selectivity. A printed Ag/AgCl electrode, which does not contain the saturated KCl/NaCl filling solution or protective layer typically found in conventional Ag/AgCl REs, was used here as a quasi‐reference electrode (q‐RE) [45]. According to the fabrication scheme, a basic Au three‐electrode pattern was initially generated on the substrate via laser ablation. Ag nanoparticles were then deposited onto the Au RE pattern through capillary printing and subsequently sintered at 200 °C to form a compact Ag layer, which was chlorinated using commercial bleach (containing approximately 2.6% (w/w) NaClO) to produce the Ag/AgCl q‐RE [46]. The WE was further functionalized with the PW_12_@MIL‐101(Cr)/PPy‐MIP using capillary printing, and all the conducting lines were finally insulated with electrical insulating tapes to avoid undesired signal contributions.

### 2.2 Material Characterization and Sensor Morphology

The structures and compositions of the PW_12_@MIL‐101(Cr)/PPy‐MIP were investigated by powder X‐ray diffraction (PXRD) and Fourier transform infrared (FT‐IR) spectroscopy, respectively, with the results presented in Figure S3. The PXRD patterns of PW_12_@MIL‐101(Cr) are consistent with literature reports, and the FT‐IR spectrum displays characteristic bands of both PW_12_ and the carboxylate group in the MIL‐101(Cr) framework, validating the successful incorporation of PW_12_ and phase purity of the material [47, 48]. After encapsulation within the polymer network, the PXRD patterns of MIL‐101(Cr) remained largely unchanged, with only slight shifts observed in certain diffraction peaks. Meanwhile, the diffraction peak intensities of PW_12_ decreased noticeably, which may be attributed to the electrostatic interaction between positively charged PPy and (PW_12_O_40_)^3‐^, thereby leading to a loss of crystallinity in PW_12_ [49]. In the FT‐IR spectrum of PW_12_@MIL‐101(Cr)/PPy‐MIP (Figure S3b), characteristic signals of PW_12_ (1077, 975, and 896 cm^−1^) and MIL‐101(Cr) (1508 and 1395 cm^−1^) can be distinctly observed, indicating their preserved structural features. Additionally, new absorption bands appear at 3112 cm^−1^ (N—H stretching vibration), 1570 cm^−1^ (C=C stretching vibration), 1214 cm^−1^ (C—N stretching vibration), and 1046 cm^−1^ (C—H in‐plane bending vibration). These bands are characteristic of the pyrrole ring in PPy [50]. Their assignments are further confirmed by the difference spectrum in Figure S3b, which excludes interference from the characteristic POM and MOF vibrational bands, thereby providing additional evidence of PPy formation. These results confirm the successful synthesis of the hybrid material of PW_12_@MIL‐101(Cr)/PPy‐MIP.

**FIGURE 1:**
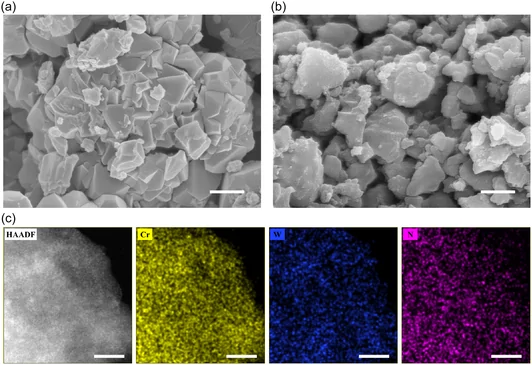
SEM images of (a) PW_12_@MIL‐101(Cr) and (b) PW_12_@MIL‐101(Cr)/PPy‐MIP, (c) HAADF‐STEM images and corresponding EDX elemental mapping of PW_12_@MIL‐101(Cr)/PPy‐MIP. Scale bar: 1 μm for (a,b); 20 nm for (c).

The surface morphologies of the synthesized materials were characterized by scanning electron microscopy (SEM). PW_12_@MIL‐101(Cr) revealed well‐defined crystalline structures with sizes of several hundred nanometers, as evidenced in Figure 1a. In contrast, PW_12_@MIL‐101(Cr)/PPy (Figure 1b for MIP and Figure S4 for nonimprinted polymer (NIP)) featured a rougher surface morphology and diminished crystallinity compared with PW_12_@MIL‐101(Cr), indicative of successful encapsulation of the PW_12_@MIL‐101(Cr) within the PPy network. Furthermore, high‐angle annular dark‐field scanning transmission electron microscopy (HAADF‐STEM) combined with energy‐dispersive X‐ray spectroscopy (EDX) elemental mapping was employed to further verify the structure of PW_12_@MIL‐101(Cr)/PPy (Figures 1c and S5a–c). In the elemental distribution maps of PW_12_@MIL‐101(Cr) and PW_12_@MIL‐101(Cr)/PPy‐NIP, the *W* signal from PW_12_ overlaps with the Cr signal from MIL‐101(Cr). The spatial distributions of W and Cr in PW_12_@MIL‐101(Cr)/PPy‐MIP are generally correlated; however, slight deviations can be observed, with W showing localized enrichment in certain regions. This phenomenon may be related to the rigorous DA removal process. MIL‐101(Cr) contains small and large cages with pentagonal windows of approximately 1.2 nm and hexagonal windows of 1.6 × 1.45 nm, respectively [47]. As the size of PW_12_ (around 1.3 nm) is smaller than the hexagonal windows of the larger cage in MIL‐101(Cr) but is larger than the pentagonal windows of smaller cages [47], partial leaching of PW_12_ from the larger cages may occur during prolonged soaking in the washing solution, leading to local variations of PW_12_ within PW_12_@MIL‐101(Cr)/PPy‐MIP. The high‐magnification HAADF‐STEM images of PW_12_@MIL‐101(Cr)/PPy‐MIP (Figure S5d) exhibited numerous bright areas within the particles, likely resulting from the stronger *W* signal. The dimensions of these bright areas are approximately 1–2 nm, which is in good agreement with the cage size of PW_12_ and the MIL‐101(Cr) framework, providing further evidence for the encapsulation of PW_12_ within the pores of the MIL‐101(Cr). The presence of a PPy network around the PW_12_@MIL‐101(Cr) particles was identified by the *N* signal. The slightly broader distribution of *N* compared to Cr in the PW_12_@MIL‐101(Cr)/PPy confirms the successful attachment of PPy on the MOF framework, as shown by the intensity profiles in Figure S5a–c.

X‐ray photoelectron spectroscopy (XPS) measurements were performed to further determine elemental composition and chemical bonding states. The XPS spectrum of MIL‐101(Cr) contains characteristic signals of Cr, O, and C (Figure S6). Compared with MIL‐101(Cr), an additional W signal was observed in the spectrum of PW_12_@MIL‐101(Cr), while both *N* and *W* signals were detected in the spectrum of PW_12_@MIL‐101(Cr)/PPy‐MIP. As shown in Figures S7–S9, the O 1s spectra can be fitted into three peaks at 533, 532, and 530 eV, which are assigned to metal oxides (O1), hydroxide/C=O groups (O2), and C—O groups (O3), respectively. The relative contribution of O1 in PW_12_@MIL‐101(Cr) and PW_12_@MIL‐101(Cr)/PPy‐MIP is higher than that in MIL‐101(Cr), indicating the incorporation of PW_12_. The C 1s spectra exhibit three typical peaks at 288.9, 286.2, and 285.0 eV, corresponding to —COOR, C—O/C—N, and C—C/C—H bonds, respectively. In PW_12_@MIL‐101(Cr)/PPy‐MIP, the proportion of C—O/C—N increased compared with the other samples, which can be attributed to the presence of C—N bonds in PPy. These findings confirm the successful encapsulation of PW_12_ and the formation of PPy in PW_12_@MIL‐101(Cr)/PPy‐MIP.

The surface area and porosity of the synthesized MIP materials play crucial roles in determining the sensing performance of the electrochemical sensor. These properties were evaluated by nitrogen adsorption–desorption measurements, and the corresponding isotherms and fitting results are presented in Figure S10 and Table S1, respectively. The Brunauer–Emmett–Teller (BET) surface area and pore volume of MIL‐101(Cr) were calculated to be 3223.59 m^2^ g^−1^ and 1.82 cm^3^ g^−1^, respectively, which are consistent with previously reported values [51, 52]. After the incorporation of PW_12_, the BET surface area and pore volume of PW_12_@MIL‐101(Cr) decreased to 1695.63 m^2^ g^−1^ and 0.73 cm^3^ g^−1^, respectively. Moreover, the PW_12_@MIL‐101(Cr)/PPy‐MIP composite still exhibited a relatively high BET surface area (1748.62 m^2^ g^−1^) and pore volume (0.74 cm^3^ g^−1^), with both values remaining comparable to those of PW_12_@MIL‐101(Cr) despite being slightly higher. This can be attributed to the partial leaching of PW_12_ from the larger cages of MIL‐101(Cr), which increased the surface area and pore volume of PW_12_@MIL‐101(Cr), whereas the imprinted polypyrrole did not completely hinder pore accessibility. In contrast, the NIP material exhibited significantly lower surface area and pore volume values of 443.79 m^2^ g^−1^ and 0.19 cm^3^ g^−1^, indicating that dense nonimprinted PPy covered the surface of PW_12_@MIL‐101(Cr) and blocked a substantial fraction of the accessible pores.

**FIGURE 2:**
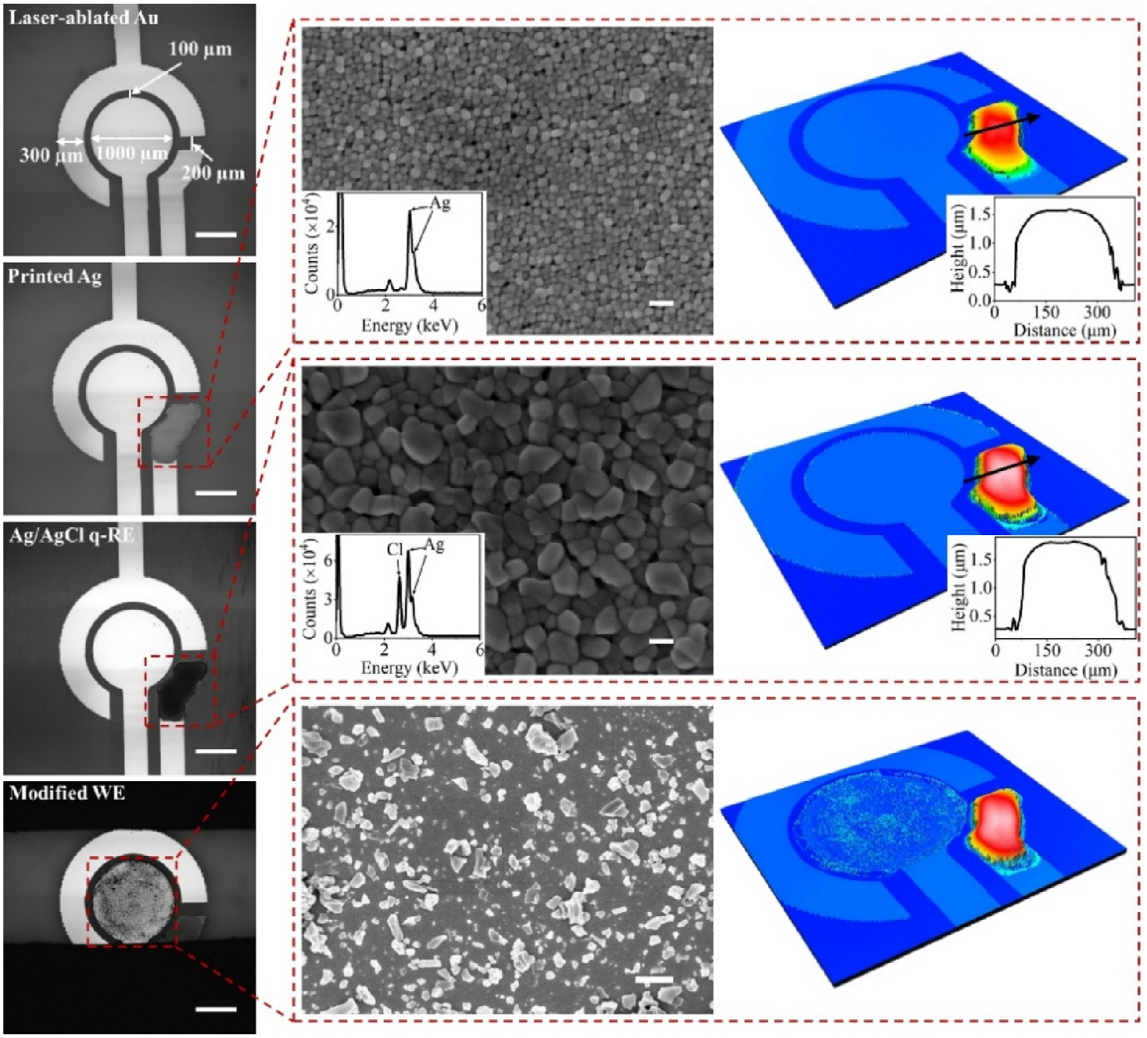
Optical microscope images (left), SEM images with corresponding EDX spectra (middle), and 3D surface morphology images with height profiles along the indicated arrow (right) of the three‐electrode systems at each fabrication stage. Scale bar: 500 μm for microscope images; 200 nm for SEM images of Ag and Ag/AgCl q‐RE; 1 μm for SEM image of modified WE.

The fabricated electrode was characterized at each stage using an optical microscope, SEM, and a 3D optical profilometer to verify the success of each step, as shown in Figure 2. The dimensions of the three‐electrode configuration were defined by the laser‐ablated Au pattern, featuring a WE diameter of 1000 and a 100 μm gap between the WE and the CE/RE. The bright metallic luster and a compact continuous nanoparticle film with particle sizes of 30–50 nm were observed for the printed Ag. EDX result (inset in the SEM image) confirmed the high purity of the Ag deposition. According to the 3D surface morphology, the printed Ag formed a uniform layer with an average thickness of approximately 1.25 μm. After chlorination with the bleach, the Ag surface lost its metallic luster and darkened due to the formation of the AgCl layer. This transformation was accompanied by an increase in particle size to over 200 nm and a thickness growth to approximately 1.5 μm. EDX verified the presence of the Cl element, with an Ag:Cl atomic ratio of 61.23%:38.77%, demonstrating the successful construction of the Ag/AgCl q‐RE. Microscopy analyses verified uniform deposition of PW_12_@MIL‐101(Cr)/PPy‐MIP on the surface of WE.

### 2.3 Electrochemical Properties of the MIP Sensor

**FIGURE 3:**
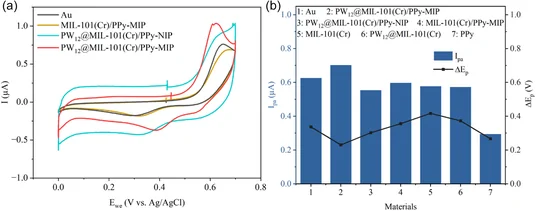
(a) CVs of electrodes incorporating different components in 1 M KCl (pH 1.0) containing 200 μM DA at the scan rate of 50 mV s^−1^. MIP: molecularly imprinted polymer; NIP: nonimprinted polymer, (b) Comparison of *I*_pa_ and Δ*E*_p_ for electrodes modified with different materials.

Cyclic voltammetry (CV), a widely used potential sweep technique for investigating the redox behavior of molecules, was then performed to evaluate the electrochemical properties of the fabricated sensors. Measurements were conducted in 1 M KCl (pH 1.0), since the PW_12_ is highly stable under acidic conditions, and the autoxidation of DA can be suppressed in an acidic environment [53, 54]. According to the CV curve of the bare Au electrodes recorded in the presence of DA (Figure S11a), the oxidation (anodic peak) of DA occurs at 0.63 V versus Ag/AgCl, with a corresponding reduction peak (cathodic peak) located at 0.29 V versus Ag/AgCl during the reverse scan. Theoretically, a reversible electrochemical redox reaction with rapid electron transfer reveals symmetrical anodic and cathodic peaks, with a peak‐to‐peak separation (Δ*E*_p_) of 59/*n* mV, where *n* is the number of electrons involved in the reaction [55, 56]. However, DA‐*O*‐quinone is prone to side reactions, such as intramolecular cyclization, which prevents complete reversibility [54]. As a result, only a portion of the DA‐*O*‐quinone can be reduced back to DA, leading to a decreased current of the cathodic peak and an increased Δ*E*_p_ [55]. Under these conditions, the anodic peak current was selected for further statistical analysis. However, a small peak at 0.64 V versus Ag/AgCl was observed for the Au electrodes in KCl solution in the absence of DA, which is attributed to the intrinsic response of the electrodes and also observed for the other modified electrodes. Therefore, the background curve of each electrode was recorded prior to DA detection and subtracted from the corresponding CV curve obtained with the same electrode in the presence of DA to eliminate background interference for subsequent data analysis. Figure S11b–g represents the CV curves of the modified electrodes in the absence and presence of DA, and Figure 3a compares the electrochemical responses of 200 μM DA at electrodes modified with different MIP/NIP materials (a comprehensive comparison of all investigated modified electrodes is provided in Figure S11h). Functionalization with PW_12_@MIL‐101(Cr)/PPy‐MIP resulted in an enhanced anodic peak current compared to the bare Au electrode. In contrast, electrodes modified with MIL‐101(Cr), PW_12_@MIL‐101(Cr), and MIL‐101(Cr)/PPy‐MIP had relatively weaker electrochemical responses toward DA. These results highlight the synergistic effect among PW_12_, MIL‐101(Cr), and PPy in improving the electrochemical detection performance for DA. The PW_12_@MIL‐101(Cr)/PPy NIP introduced a higher non‐Faradaic current, likely due to the denser PPy network. The anodic peak current after background subtraction (*I*_pa_, with *I*_pa_ *’* denoting the current before subtraction) and Δ*E*_p_ were calculated and are plotted in Figure 3b. Among the tested modified electrodes, the PW_12_@MIL‐101(Cr)/PPy‐MIP‐based electrode demonstrated good electrochemical performance, showing the highest *I*_pa_ and the smallest Δ*E*_p_. The decreased Δ*E*_p_ value of the PW_12_@MIL‐101(Cr)/PPy‐MIP electrode indicates that the PW_12_@MIL‐101(Cr)/PPy‐MIP composite effectively facilitated the electrochemical oxidation of DA and enhanced the charge transfer rate. Moreover, upon extending the potential window toward a more negative direction, a significant pair of redox peaks, consisting of an anodic peak at 0.07 V versus Ag/AgCl and a cathodic peak at −0.24 V versus Ag/AgCl, appeared in the CV curve of the PW_12_@MIL‐101(Cr)/PPy‐MIP electrode in the absence of DA (Figure S11i). These peaks are likely attributed to the one‐electron redox process of PW_12_, suggesting that PW_12_ is electrochemically active under the measurement conditions [57, 58]:

$${\left({\mathrm{PW}}_{12}O_{40}\right)}^{3‐}\;+\;e^{‐}\;↔\;{\left({\mathrm{PW}}_{12}O_{40}\right)}^{4‐}$$

The Δ*E*_p_ of PW_12_ was calculated to be 310 mV, suggesting a quasi‐reversible process. Such behavior is generally associated with sluggish electron‐transfer kinetics and/or uncompensated iR drop, which can be affected by electrolyte concentration and composition, scan rate, and electrode interface properties [59, 60, 61].

### 2.4 Optimization of Experimental Parameters

**FIGURE 4:**
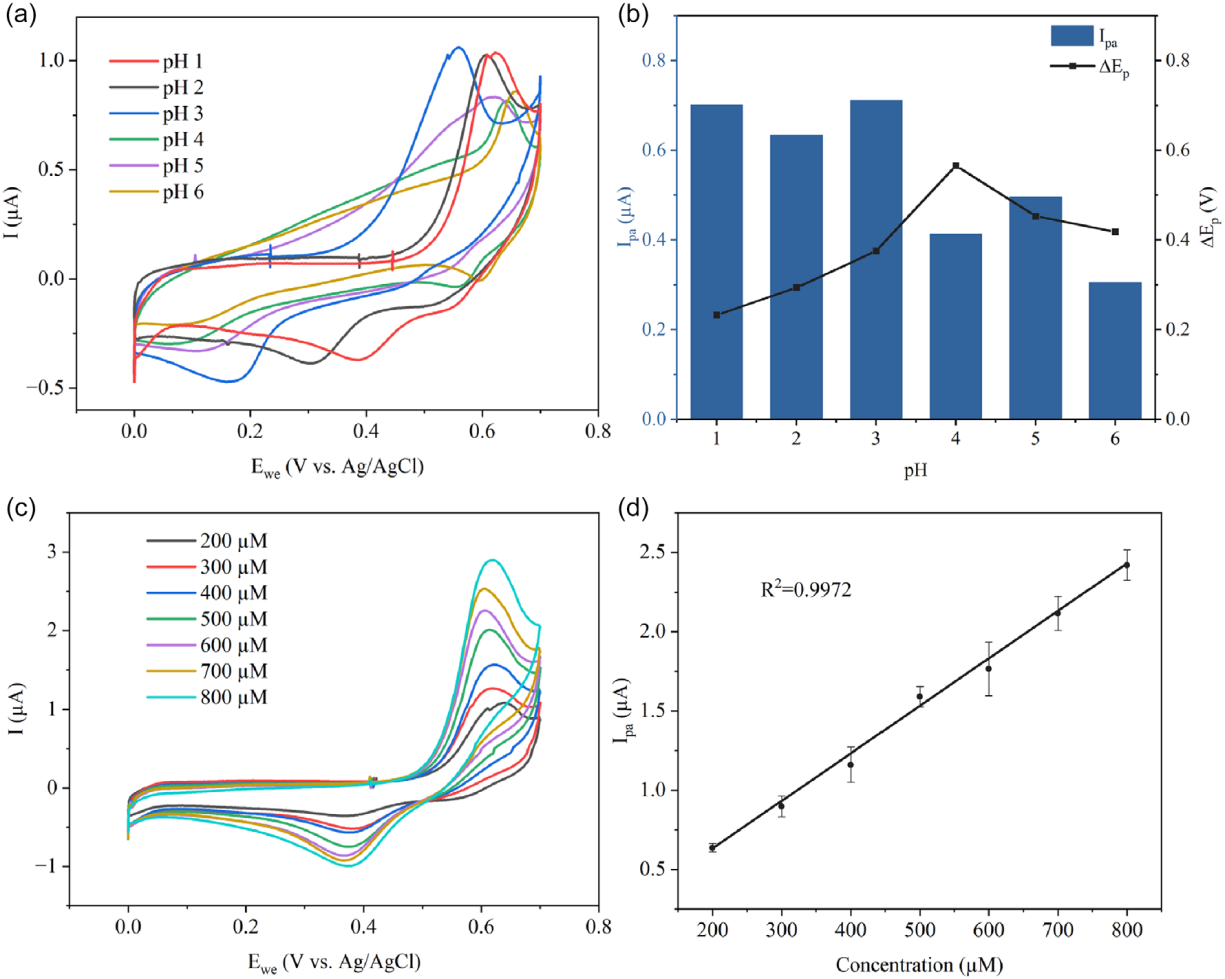
(a) CVs of the PW_12_@MIL‐101(Cr)/PPy‐MIP electrochemical sensor in 1 M KCl at various pH values. Scan rate: 50 mV s^−1^; DA concentration: 200 μM, (b) Corresponding *I*_pa_ and Δ*E*_p_ extracted from (a), (c) CV curves of the PW_12_@MIL‐101(Cr)/PPy‐MIP electrochemical sensor in 1 M KCl (pH 1.0) with DA concentrations ranging from 200 to 800 μM. Scan rate: 50 mV s^−1^, (d) Corresponding calibration plot of *I*_pa_ versus *C*_DA_ for (c).

Since the electrochemical behavior of DA is pH‐dependent, *I*_pa_ and Δ*E*_p_ may vary with changes in pH [62]. Additionally, the electrochemical properties of PW_12_ are also influenced by the pH [58]. In the pH range of 2.0–7.0, PW_12_ tends to decompose into its lacunary form, PW_11_, which exhibits distinct electrochemical characteristics [53]. Therefore, the effect of pH on the electrochemical response of PW_12_@MIL‐101(Cr)/PPy‐MIP sensor toward DA was explored, and the results are presented in Figure 4a,b. In the pH range of 1.0–3.0, the sensors displayed similar *I*_pa_ values, with a slight increase in Δ*E*_p_ observed as the pH increased. Meanwhile, the anodic peak potential (*E*_pa_) shifted negatively, suggesting the involvement of protons in the electrode reaction [63]. However, as the pH increased further to 4.0–6.0, the CV curves became asymmetrical, indicating a loss of electrochemical reversibility. This irreversible behavior of the DA redox reaction at higher pH may result from the enhanced kinetics of DA autoxidation and the intramolecular cyclization of DA‐*O*‐quinone [54]. Therefore, the pH 1.0 KCl solution was employed in subsequent analytical experiments. Although the current operating pH remains far from the physiological conditions, the hydrolysis of PW_12_ to PW_11_ in aqueous solution is influenced not only by pH, but also by the concentration and the composition of the buffer, both of which can facilitate the stabilization of PW_12_ at a relatively higher pH [64]. Hence, the operational pH range could be modestly extended in future studies through careful optimization of the buffer concentration and buffer composition. In addition, various POMs exhibit higher pH tolerance, such as [V_3_O_10_]^5−^ and [V_4_O_13_]^6−^, making them promising alternatives for applications under neutral pH conditions [65].

The monomer‐to‐template ratio in the MIP matrix influences the MIP structure, its affinity for template molecules, and the number of active imprinted cavities available for analyte binding, thereby affecting the electrochemical performance [66]. The *I*_pa_ values for 200 μM DA on modified sensors with monomer‐to‐template mole ratios of 4:1, 3:1, and 2:1 are shown in Figure S12a. The highest *I*_pa_ occurred at the 4:1 ratio. At high DA concentrations during polymerization, the pyrrole monomer cannot efficiently react with DA molecules in solution, resulting in a portion of monomers failing to form a stable polymer. Meanwhile, excess free DA may interfere with polymerization, causing disorder within the polymer matrix structure and consequently influencing electron transfer efficiency. Therefore, a monomer‐to‐template mole ratio of 4:1 was selected.

The morphology of the modification layer on the WE, determined by the quality of the printing process, plays a critical role in the electrochemical properties of the system [56]. Accordingly, the electrochemical responses toward DA were evaluated on MIP sensors with different printed PW_12_@MIL‐101(Cr)/PPy‐MIP layers on the WE. The *I*_pa_ values for 200 μM DA reached the maximum when two layers of the PW_12_@MIL‐101(Cr)/PPy‐MIP composite were printed (Figure S12b), while further increases in layer number led to a decline in *I*_pa_, likely due to excessive thickness and surface roughness from multilayer printing hindering the electron transfer efficiency.

### 2.5 Performance of the MIP Sensor for DA Detection

Under an optimized pH environment, the determination of DA was performed using a PW_12_@MIL‐101(Cr)/PPy‐MIP electrochemical sensor in 1 M KCl. The CV responses for the determination of DA over a concentration range of 200–800 μM and the calibration plots are depicted in Figure 4c,d, respectively. At all tested concentrations, the shape of the CV curves and the *E*_pa_ and cathodic peak potentials (*E*_pc_) remained unchanged, while the anodic and cathodic peak currents increased proportionally with the DA concentration. The calibration plot of *I*_pa_ versus DA concentration exhibits a good linear relationship, described by the regression equation *I*_pa_ (μA) = 0.003*C* (μM) + 0.03409, with a correlation coefficient (*R* ^2^) of 0.9972. The limit of detection (LOD) and limits of quantification (LOQ) for DA were calculated as 59.03 and 178.87 μM, respectively, according to the calibration plot. The comparison of the sensing performance of the PW_12_@MIL‐101(Cr)/PPy‐MIP sensor with other reported composite‐based DA sensors is presented in Table S2. Although the PW_12_@MIL‐101(Cr)/PPy‐MIP electrochemical sensor currently lacks sufficient sensitivity to directly detect DA in biological samples at physiological concentrations (0.01–1 μM) [67], this limitation, which arises not only from the material or electrode modification itself but also from the electrode configuration, measurement parameters, and experimental conditions [56, 68], can be overcome through appropriate enhancements in electrode design. Nevertheless, the sensor displays a strong linear response at higher concentrations, indicating the potential of the PW_12_@MIL‐101(Cr)/PPy‐MIP composite for electrochemical detection of DA in biofluids when combined with appropriate sample pretreatments, such as preconcentration or resuspension of lyophilized samples.

**FIGURE 5:**
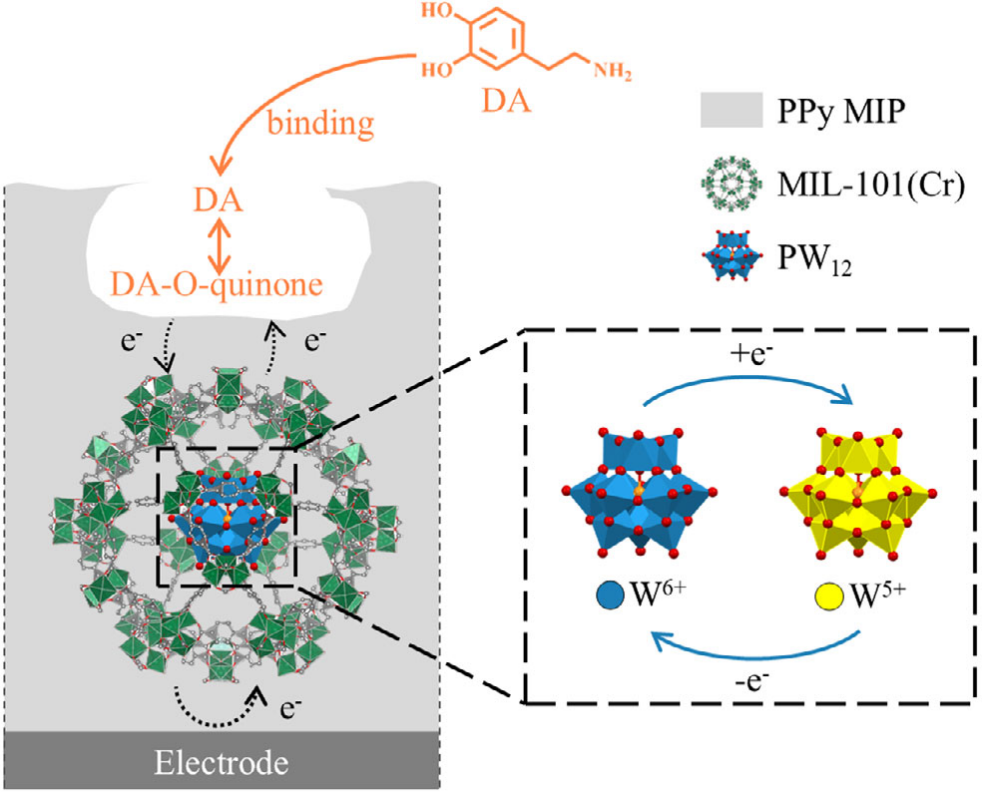
Schematic illustration of the possible mechanism for the DA detection using PW_12_@MIL‐101(Cr)/PPy‐MIP sensor.

Additionally, CVs were recorded in the 1 M KCl (pH 1.0) solution containing 200 μM DA using the PW_12_@MIL‐101(Cr)/PPy‐MIP sensor at various scan rates (Figure S13a). The *I*_pa_ increased linearly with scan rate, accompanied by a slight positive shift in the *E*_pa_. A strong linear relationship was observed (Figure S13b) with *R* ^2^ of 0.9921, demonstrating that the electron transfer process of DA at the PW_12_@MIL‐101(Cr)/PPy‐MIP sensor was controlled by surface adsorption [69]. Hence, the possible sensing mechanism of the PW_12_@MIL‐101(Cr)/PPy‐MIP can be proposed as follows (illustrated in Figure 5): DA molecules were adsorbed and enriched within the PPy matrix through interactions with the specific binding cavities, where the redox reaction of DA occurred. PPy enhanced the conductivity of the composite and facilitated electron transfer. Meanwhile, the large surface area and porous structure of MIL‐101(Cr) allowed electrolyte ions to penetrate the framework and form an electrochemical double layer in favor of charge transport. The charge acceptance and donation processes occurred with PW_12_ confined within the framework via the transformation of *W* (*W* ^6+^ ↔ *W* ^5+^). Despite the limited transfer kinetics, this process nevertheless promoted charge transfer throughout the MOF, as evidenced by the comparison of the electrochemical properties of MIL‐101(Cr)/PPy‐MIP and PW_12_@MIL‐101(Cr)/PPy‐MIP shown in Figure 3b.

### 2.6 Selectivity of the MIP Sensor

**FIGURE 6:**
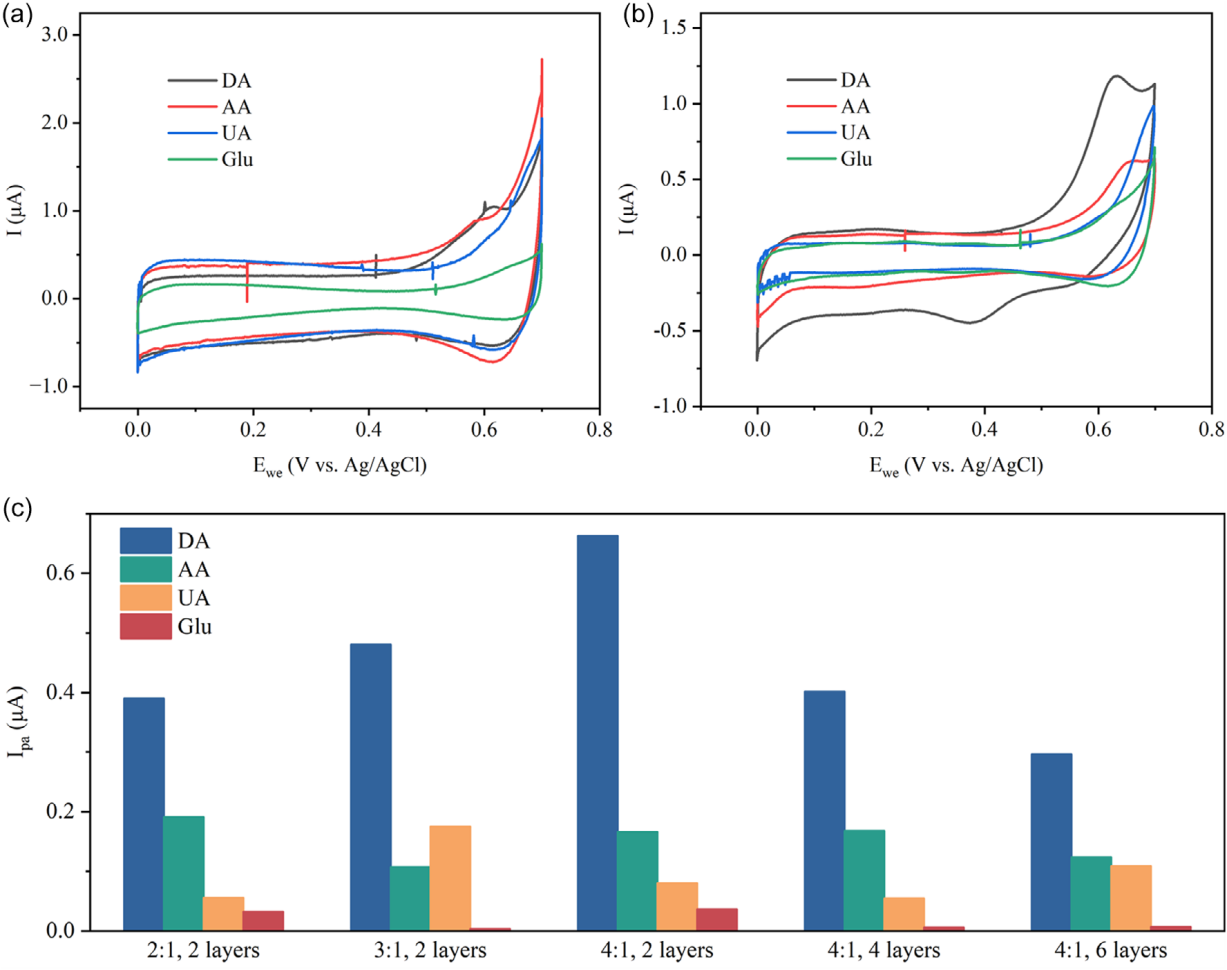
CVs of (a) the PW_12_@MIL‐101(Cr)/PPy‐NIP sensors and (b) the PW_12_@MIL‐101(Cr)/PPy‐MIP sensors in 1 M KCl (pH 1.0) containing 200 μM DA/AA/UA/Glu, (c) Electrochemical responses of 200 μM DA/AA/UA/Glu at 0.63 V versus Ag/AgCl on PW_12_@MIL‐101(Cr)/PPy‐MIP sensors with different monomer‐to‐template mole ratios or number of printed layers.

Ascorbic acid (AA) and uric acid (UA) are two common electroactive interferents that coexist with DA in biological fluids. Their presence greatly complicates the electrochemical detection of DA, particularly since AA is typically present at higher concentrations and undergoes oxidation at a potential similar to that of DA [70]. Glucose (Glu) is also considered a potential interferent in DA detection because it coexists with DA in blood and can be electrochemically detected. Therefore, the selectivity of the prepared PW_12_@MIL‐101(Cr)/PPy‐MIP sensors was evaluated toward DA, AA, UA, and Glu at the same concentration (200 µM). Figure 6a reveals that the NIP sensors failed to distinguish between DA and AA as their anodic peaks were nearly overlapping and their *I*_pa_ *’* were similar. Within the measured potential range, AA produced a response exclusively at the *E*_pa_, while UA and Glu generated no measurable signals in either anodic or cathodic regions. However, for the PW_12_@MIL‐101(Cr)/PPy‐MIP sensor (Figure 6b), the current response toward DA exceeded that of AA, whereas AA exhibited a slightly higher *E*_pa_ than DA. Notably, similar to the NIP sensor, no significant signals were observed for UA and Glu. The imprinting factor, defined as the ratio of analyte binding on the MIP to the NIP ((signal of MIP)/(signal of NIP)), is commonly used to evaluate imprinting efficiency and is expected to be greater than one for successful imprinting [71]. In this work, the imprinting factor ((*I*_pa_ of MIP)/(*I*_pa_ of NIP), the specific values are shown in Table S3) for PW_12_@MIL‐101(Cr)/PPy‐MIP was calculated to be 2.56, indicating a specific recognition ability of the synthesized material toward the DA.

The effects of varying the monomer‐to‐template mole ratios or number of printed MIP layers on sensor selectivity were also evaluated, as illustrated in Figure 6c. The responses of the four analytes were recorded at 0.63 V versus Ag/AgCl, corresponding to the *E*_pa_ of DA. The selectivity of DA on the PW_12_@MIL‐101(Cr)/PPy‐MIP sensor varied with changes in the properties and thickness of the modifier on the WE, while these factors had minimal impact on responses to AA, UA, and Glu. With a monomer‐to‐template ratio of 4:1 and two layers of material deposited on the WE, the MIP sensor displayed optimal selectivity, with responses to AA, UA, and Glu accounting for only 25.07%, 12.14%, and 5.56% of the response to DA, respectively, highlighting the potential of the PW_12_@MIL‐101(Cr)/PPy‐MIP to enhance the selectivity of electrochemical DA detection. The partial responses toward AA and UA suggest that the selectivity of the current PW_12_@MIL‐101(Cr)/PPy‐MIP system remains governed by a combination of imprinting‐specific recognition and residual nonspecific electrochemical contributions, and further optimization of the imprinting process and fabrication quality is therefore expected to improve the discrimination between the target and interferents.

### 2.7 Stability, Regeneration Ability, and Reproducibility of the MIP Sensor

**FIGURE 7:**
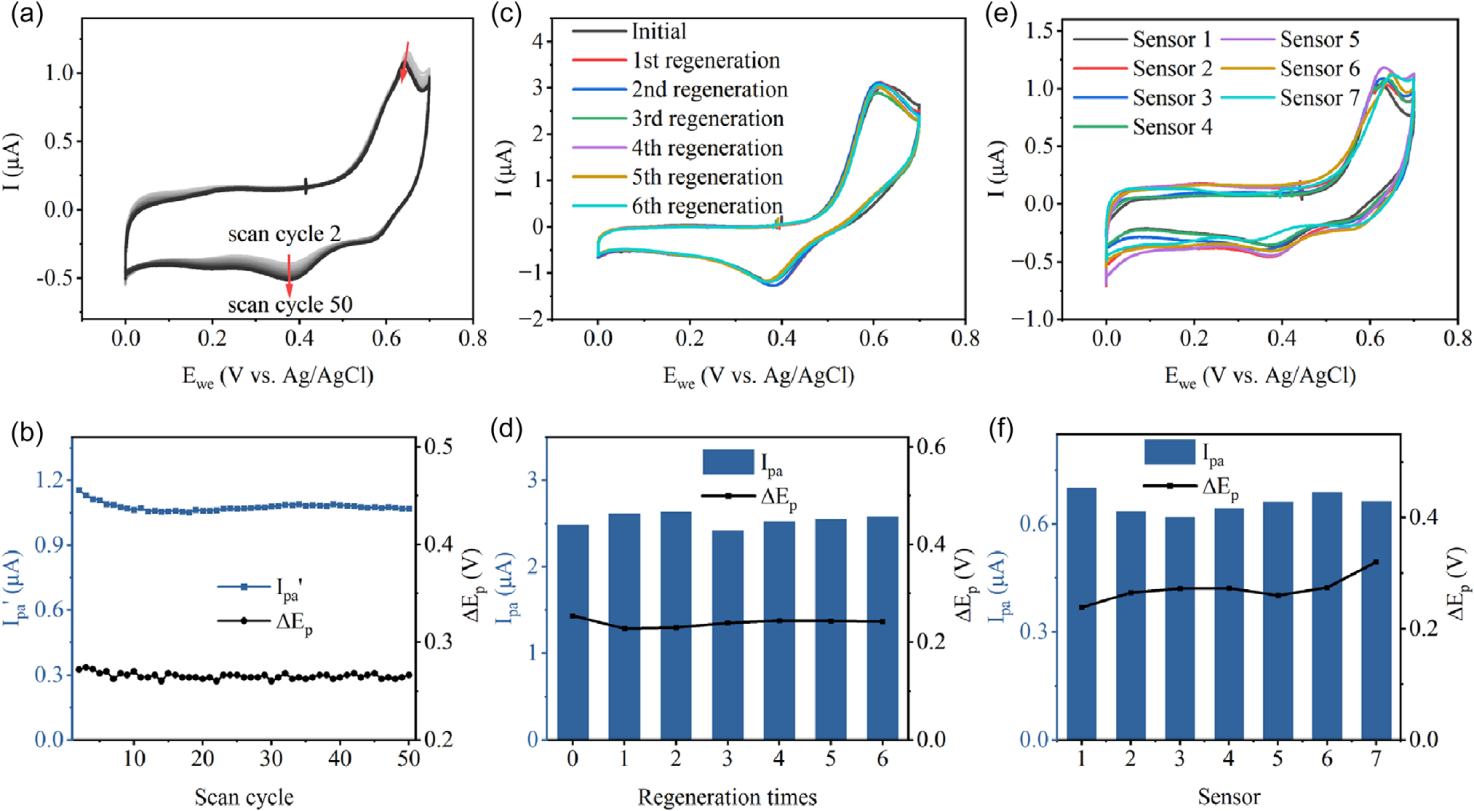
Comparison of the MIP sensor performance: (a) CV curves and (b) peak currents illustrating stability over 50 consecutive scan cycles (*C*_DA_ = 200 µM); (c) CV responses and (d) *I*_pa_ illustrating regeneration ability of fabricated sensor over six rebinding‐washing cycles (*C*_DA_ = 800 µM); (e) CVs and (f) *I*_pa_ illustrating reproducibility across seven independent sensors (*C*_DA_ = 200 µM).

The stability, regeneration ability, and reproducibility of an electrochemical sensor are critical parameters for its practical analytical applications. To investigate the short‐term stability of the PW_12_@MIL‐101(Cr)/PPy‐MIP sensor, CV measurements were performed under 200 μM DA in 1 M KCl (pH 1.0) over 50 consecutive scan cycles. Since electrochemical systems typically do not reach equilibrium immediately during the initial scan, the results presented begin from the second scan cycle (Figure 7a,b). The CV curves remained consistent in shape throughout the scanning process, demonstrating stable electrochemical behavior of the sensor. As the number of scan cycles increased, the anodic peak current showed a slight decrease, while the cathodic peak current increased correspondingly. Specifically, *I*_pa_ *’* decreased by only 8.05% within the first ten cycles and then stabilized in the subsequent cycles. In addition, the Δ*E*_p_ stayed unchanged during the entire measurement. These observations confirm that the PW_12_@MIL‐101(Cr)/PPy‐MIP sensor possessed satisfactory stability during the measurement. Furthermore, the MIP sensor can be regenerated by removing the template molecules through successive washing with isopropanol and water, allowing it to be reused for another determination. As shown in Figure 7c,d, after exposure to a high concentration of DA (800 μM) over six rebinding‐removal cycles using a single sensor, no significant change in the current responses to DA was observed, indicating that the PW_12_@MIL‐101(Cr)/PPy‐MIP sensor retains its functionality and can be reused effectively for at least six regeneration cycles. Figure S14 shows the morphology of PW_12_@MIL‐101(Cr)/PPy‐MIP on the WE after the stability and regeneration test, with no obvious morphological changes observed compared to the pristine sensor (Figure 2). This result further confirms the structural stability of the synthesized hybrid material, as well as the functional stability and regeneration capability of the fabricated sensors. In addition to stability and regeneration ability, the reliability of printed sensors is also closely associated with their reproducibility. To assess this parameter, CV responses to DA in 1 M KCl (pH 1.0) solution were recorded using seven PW_12_@MIL‐101(Cr)/PPy‐MIP sensors that were fabricated independently under identical experimental conditions. All MIP sensors demonstrated similar electrochemical behavior, with consistent *I*_pa_ and Δ*E*_p_ (Figure 7e,f). The mean *I*_pa_ value of seven MIP sensors was 0.66 μA, with a relative standard deviation of 4.09%, confirming that the fabricated MIP sensor has excellent reproducibility.

## 3 Conclusion

In summary, a sensitive and selective electrochemical sensor for DA detection was fabricated using a PW_12_@MIL‐101(Cr)/PPy‐MIP hybrid‐modified three‐electrode system. The PW_12_@MIL‐101(Cr)/PPy‐MIP sensor exhibited good recognition capacity for DA, along with excellent stability, regeneration ability, and reproducibility, and showed a linear relationship between DA concentration and current response in CV with a LOD of 59.03 μM. The good discrimination ability of the MIP sensor toward DA was proven by its capability to distinguish DA from AA, UA, and Glu. These findings highlight the synergistic advantages of POMs, MOFs, and MIPs in electrochemical sensing and the potential of the fabricated MIP sensor for DA measurement. Future work will focus on fabricating various POM@MOF/MIP hybrid materials with higher sensitivity and selectivity, as well as optimizing sensor configurations, both of which are expected to improve the performance of electrochemical sensors.

## 4 Experimental Section

### 4.1 Reagents and Chemicals

Sodium tungstate dihydrate (Na_2_WO_4_·2H_2_O, ≥99%), disodium hydrogen phosphate dihydrate (Na_2_HPO_4_·2H_2_O, ≥99%), chromium nitrate nonahydrate (Cr(NO_3_)_3_·9H_2_O, 99%), 1,4‐benzenedicarboxylate (BDC, 98%), NaOH (≥98%), pyrrole (98%), DA hydrochloride (≥98%), UA (≥99%), AA (≥99%), Glu (≥99.5%), iron(III) chloride hexahydrate (FeCl_3_·6H_2_O, ≥98%), commercial available silver nanoparticle ink (Sigma–Aldrich, particle ≤50 nm, 30–35 wt% in triethylene glycol monoethyl ether), DMSO (≥99.9%), KCl (99%), and HCl (37%) were purchased from Sigma–Aldrich (Germany). Ethanol (99.98%) was obtained from VWR (Germany). Distilled water was produced in the laboratory using an Arium Pro system (Sartorius, Germany).

### 4.2 Synthesis of H_3_[PW_12_O_40_] (PW_12_)

PW_12_ was obtained according to the reported procedure [72]. Generally, 6.48 g (19.64 mmol) Na_2_WO_4_·2H_2_O and 1.04 g (5.84 mmol) Na_2_HPO_4_·2H_2_O were dissolved in 10 mL boiling water with stirring to get a clear solution. Subsequently, 5.2 mL of HCl solution was added dropwise to the above solution under stirring, leading to a white precipitate. After cooling the suspension in an ice bath, the white precipitate was collected by filtration and quickly washed with cold water to remove residual impurities.

### 4.3 Synthesis of PW_12_@MIL‐101(Cr)

PW_12_@MIL‐101(Cr) was prepared using the one‐pot method following the published protocol in Ref. [47] with slight modifications [47]. Thus, a dispersion containing 5 g (12.5 mmol) Cr(NO_3_)_3_·9H_2_O, 2.08 g (12.5 mmol) BDC, and 3 g (1.04 mmol) PW_12_ was prepared in 60 mL of distilled water with stirring, followed by the addition of 1 M NaOH solution to adjust the pH to 3.0. The mixture was sealed in a Teflon‐lined autoclave (100 mL) and kept at 180 °C for 24 h. The resulting green product was thoroughly washed with ethanol and water. The synthesis process of MIL‐101(Cr) was the same as the aforementioned method, but without the addition of PW_12_ and pH adjustment.

### 4.4 Synthesis of PW_12_@MIL‐101(Cr)/PPy Composite

The PW_12_@MIL‐101(Cr)/PPy‐MIP composite was prepared by mixing 0.1 mL pyrrole, 68.33 mg DA, and 100 mg as‐synthesized PW_12_@MIL‐101(Cr) in 30 mL of 1 M HCl solution under vigorous stirring with a stirring bar (800–1000 rpm), subsequently adding FeCl_3_/HCl (0.4 g/5 mL) solution dropwise. The black product formed gradually from the suspension, which was kept at 8 °C overnight to ensure the complete polymer formation after 2 h of stirring. The final solid was washed with distilled water and ethanol until all the DA templates in the polymer network were removed. The PW_12_@MIL‐101(Cr)/PPy NIP was synthesized using the same procedure but without the addition of DA.

### 4.5 Electrode Fabrication

The base gold three‐electrode structure was laser ablated on a gold‐coated glass slide (gold thickness: 100 nm, Sigma–Aldrich) using a TruMicro 5000 ps laser (Trumpf, Germany) and, afterward, cleaned with chloroform, ethanol, and water with 30 min of sonication for each solvent. The silver nanoparticle ink was patterned on the electrode surface by capillary printing in an NLP 2000 system (Nanoink, USA) equipped with a glass capillary. The glass capillary was prepared with a fine tip using a pipette puller, and the tip was cut to obtain an opening diameter of 100–200 µm. The capillary tip was then immersed in the ink, allowing the ink to be drawn into the tip via the capillary effect. Upon contact with the substrate, the ink was transferred from the tip, resulting in the deposition of the Ag nanoparticles. The obtained Ag layer was exposed to bleach for 1 min to form the Ag/AgCl RE. Subsequently, the WE was modified via capillary printing with the PW_12_@MIL‐101(Cr)/PPy hybrid material, which was dispersed in water (4 mg mL^−1^) containing 10% DMSO. Finally, the conducting lines were covered with electrical insulating tape, and the sensors were rinsed with water and stored in a dark condition until further use.

### 4.6 Characterization

PXRD analyses were carried out on a D8 Advance X‐ray diffractometer (Bruker, USA) using a Cu Kα radiation source (*λ *= 1.54056 Å). FT‐IR spectra were recorded with a NicoletTM iS50 spectrometer (Thermo Scientific, USA) equipped with an ATR accessory. SEM images were acquired with a Leo Gemini 1530 scanning electron microscope (Carl Zeiss, Germany) operated at 5 kV after coating the samples with a 10.3 nm layer of gold using a 108 auto sputter coater (Cressington, UK). STEM images were acquired using a HAADF detector (Fischione, USA) on a Themis‐300 (Thermofisher Scientific, USA) transmission electron microscope, equipped with a probe aberration corrector and Super‐X EDX detector. The powder specimens were directly dispersed on lacy carbon‐coated Au TEM grids, and the microscope was operated at an accelerating voltage of 300 kV. STEM EDX spectrum imaging was acquired, and maps of the relevant elements were extracted using Velox software (Thermofisher Scientific, USA). 3D surface morphology was analyzed using a Profilm3D optical profilometer (Filmetrics, USA) employing white‐light interferometry (WLI), and optical microscope images were captured with an Eclipse 80i upright microscope (Nikon, Japan). Cyclic voltammograms were recorded in 1 M KCl solution over a potential range of 0 to 0.7 V under optimized conditions (pH 1.0, scan rate 50 mV s^−1^) using a VMP3 Multichannel Potentiostat (Biologic, France). The XPS measurements were carried out with a photoelectron spectrometer (Thermo VG Scientific, USA). Monochromatic Al‐K_α_ X‐rays were used for the measurements (Al *K*_α_, *hν* = 1486.6 eV). The basic pressure of the measuring chamber is typically approx. 2 × 10^−9^ mbar. The N_2_ adsorption–desorption isotherms at 77 K were measured on a 3Flex adsorption analyzer (Micromeritics, USA) after the samples were degassed overnight at 100 °C.

## Funding

This study was supported by the China Scholarship Council (No. 202106240010), Helmholtz Research Program Materials System Engineering (MSE), Deutsche Forschungsgemeinschaft (DFG) (EXC2082/1‐390761711).

## Conflicts of Interest

The authors declare no conflicts of interest.

## Supporting information

Supplementary Material

## Data Availability

The data that support the findings of this study are available from the corresponding author upon reasonable request.
