# Supplementary material for: Polyoxometalate@Metal–Organic Framework‐Based Imprinted Electrochemical Sensor for Dopamine Detection
Source: Small Sci. 2026 Aug 21;6(8):e70380. doi: 10.1002/smsc.70380 (PMC13517000; doi:10.1002/smsc.70380)
Supplement: Supplementary file 1 [file SMSC-6-e70380-s001.pdf]

Supporting Information

Polyoxometalate@Metal-Organic Framework-Based Imprinted Electrochemical Sensor for Dopamine Detection

*Wenjing Wang [1,2], Di Wang [1,2], Srivatsan K. Vasantham [1,2], Hongrong Hu [1], Yan Liu [1], Andreas H. Schäfer [3], George Mathew [1,2], Navid Hussain [1,2], Masooma Ibrahim [1,4], Jasmin Aghassi-Hagmann [1], Annie K. Powell [1,4,5], Michael Hirtz [1,2,]**

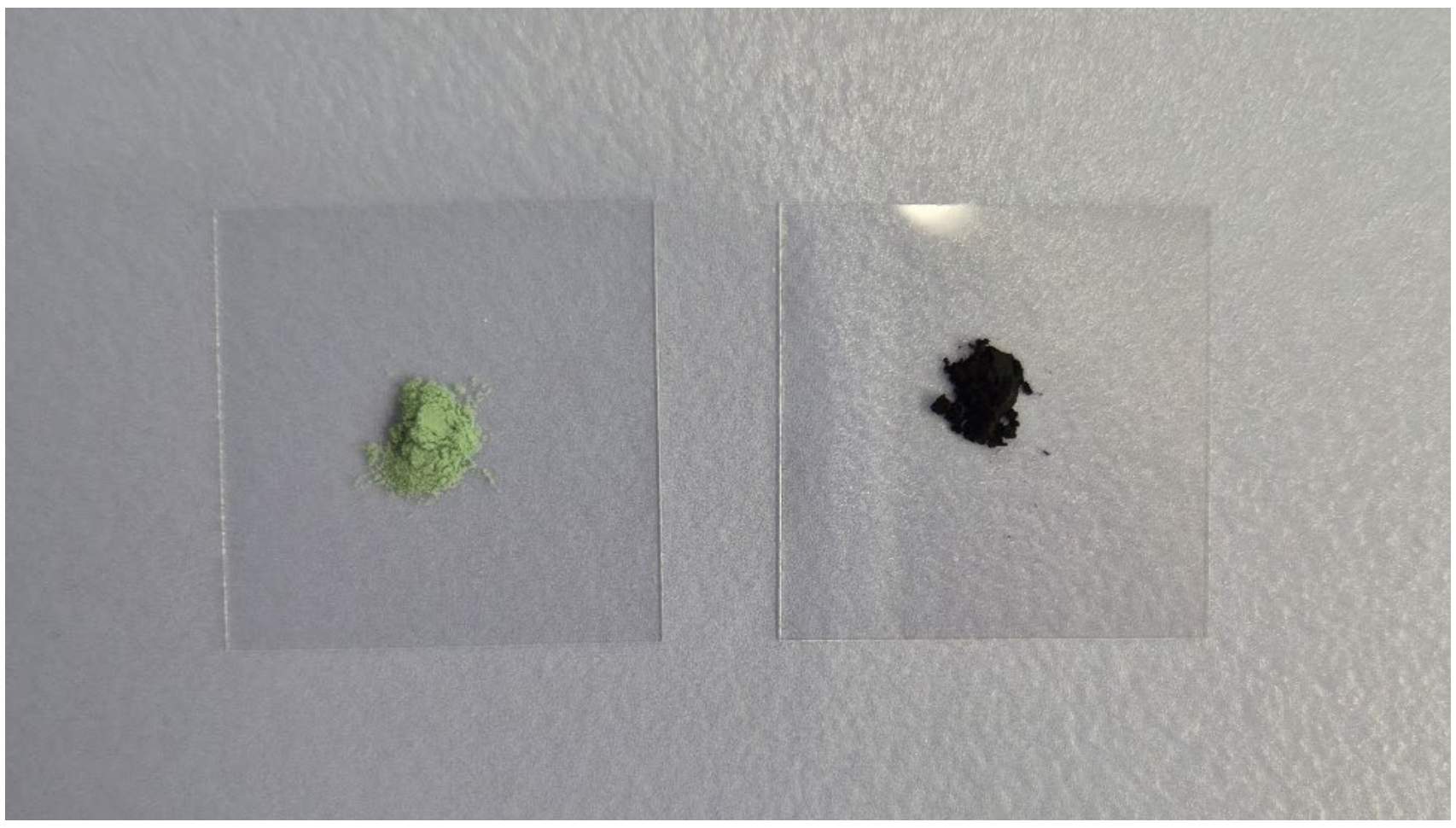

Figure S1. Photograph of PW_{12}@MIL-101(Cr) (left) and PW_{12}@MIL-101(Cr)/PPy-MIP (right).

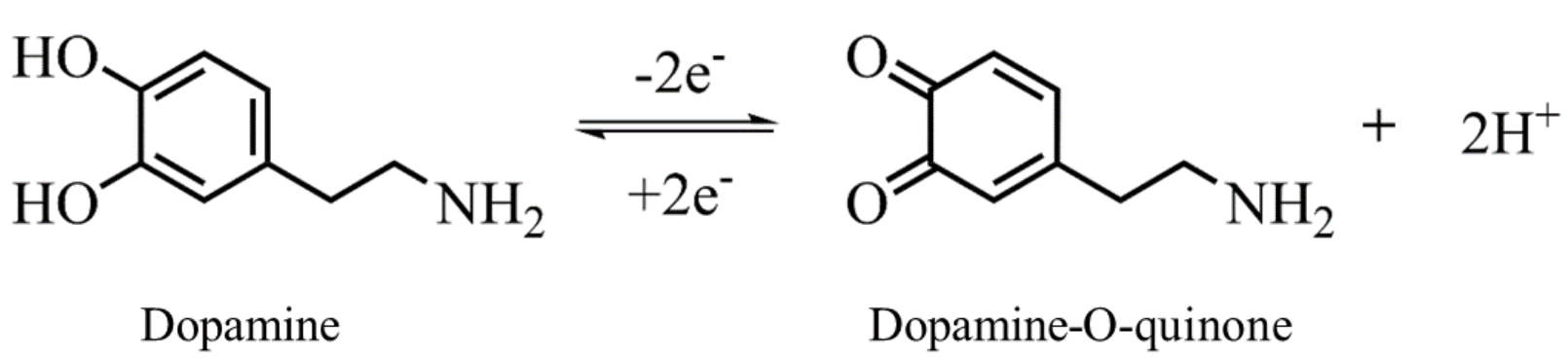

Figure S2. Redox reaction of DA.

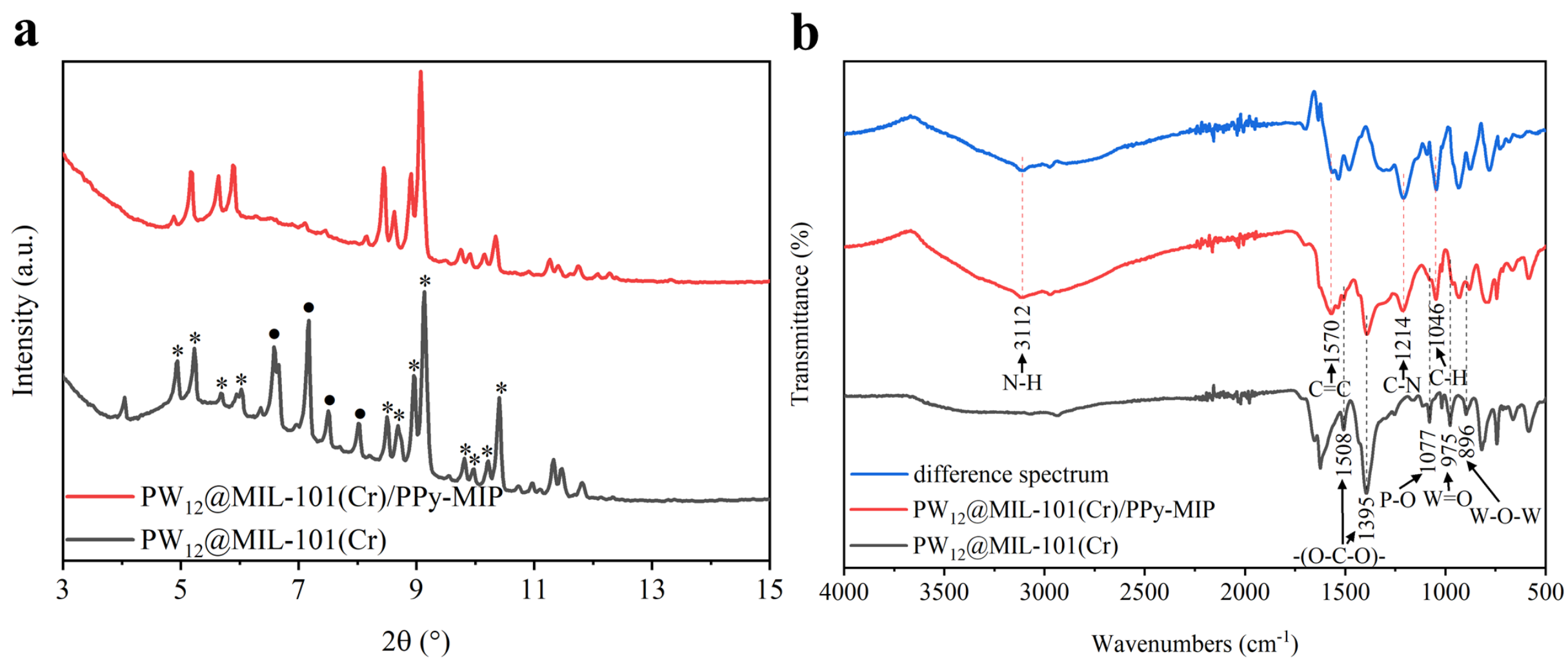

Figure S3. (a) PXRD patterns (*: MIL-101(Cr), ●: PW_{12}) and (b) FT-IR spectra of PW_{12}@MIL-101(Cr)/PPy-MIP.

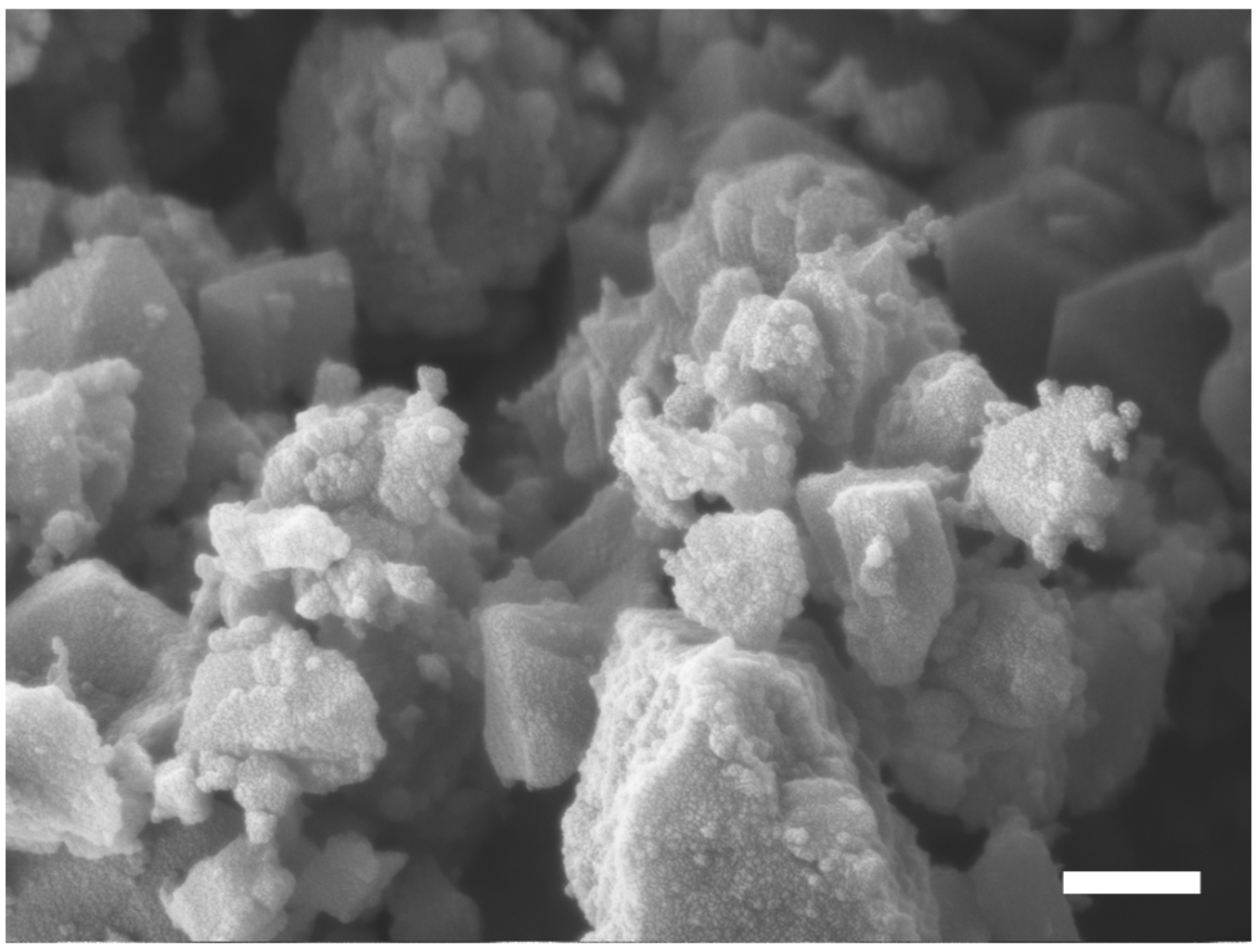

Figure S4. SEM images of PW_{12}@MIL-101(Cr)/PPy-NIP. NIP: non-imprinted polymer. Scale bar: 900 nm.

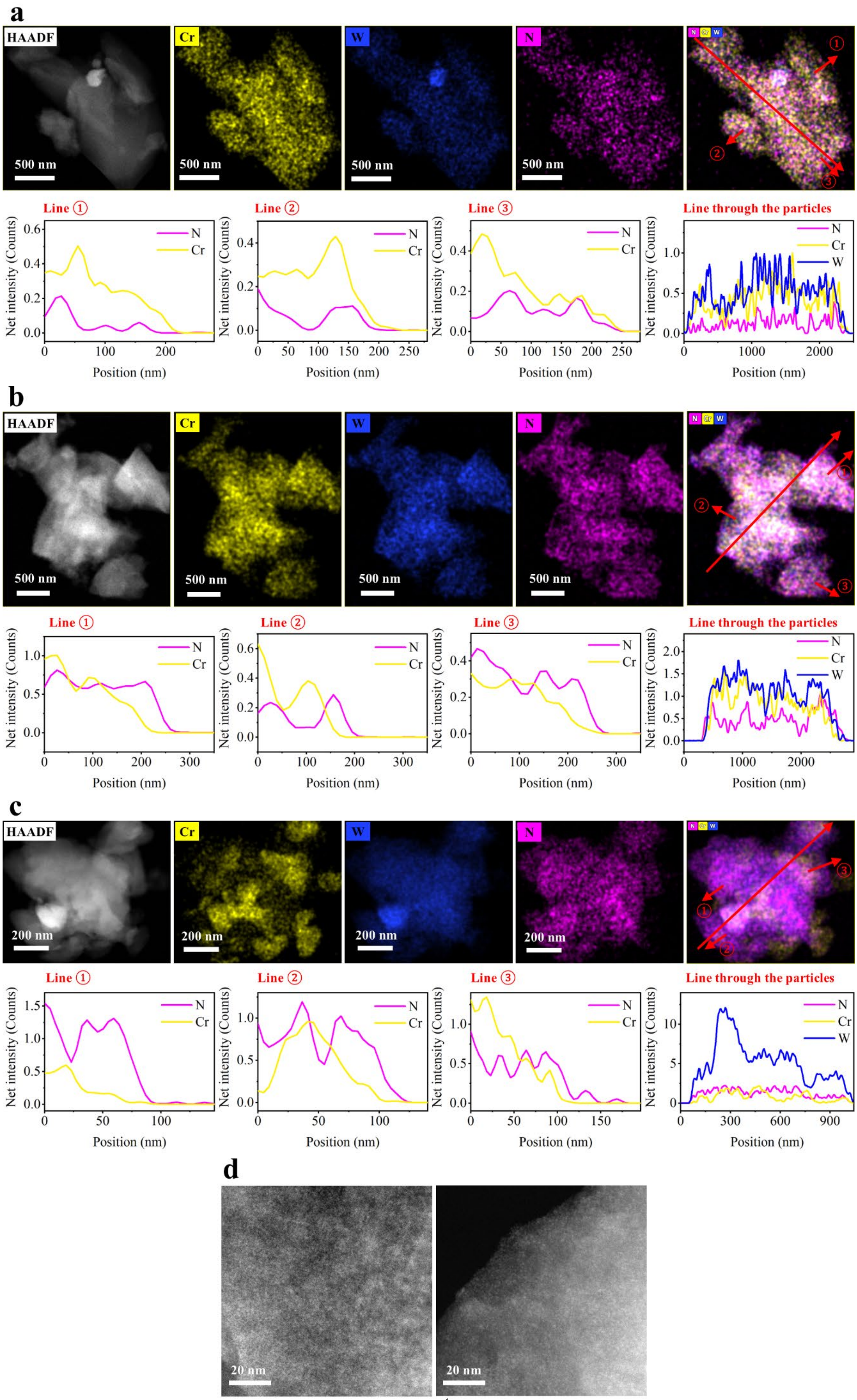
a
HAADF
Cr
W
N
500 nm
Line ①
Line ②
Line ③
Line through the particles
Net intensity (Counts)
Position (nm)
b
c
200 nm
d
20 nm

Figure S5. STEM images and corresponding EDX elemental mapping of (a) PW_{12}@MIL-101(Cr), (b) PW_{12}@MIL-101(Cr)/PPy-NIP, and (c) PW_{12}@MIL-101(Cr)/PPy-MIP, with intensity profiles extracted along the indicated arrows on the N/Cr/W merged images. The low N signal in the PW_{12}@MIL-101(Cr) may be attributed to the air. (d) STEM images of PW_{12}@MIL-101(Cr)/PPy-MIP at 980,000× magnification.

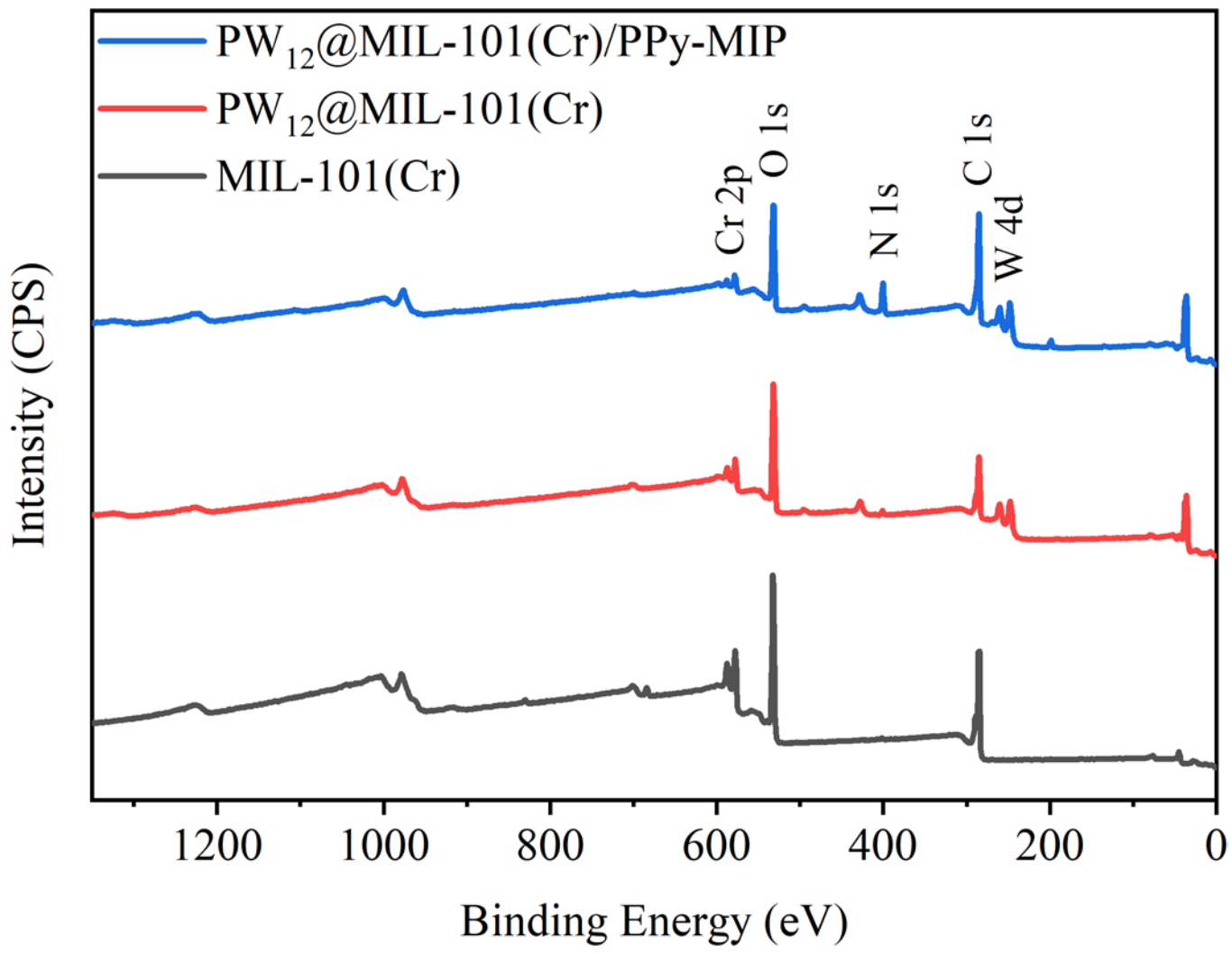

Figure S6. XPS spectra of MIL-101(Cr), PW_{12}@MIL-101(Cr), and PW_{12}@MIL-101(Cr)/PPy-MIP.

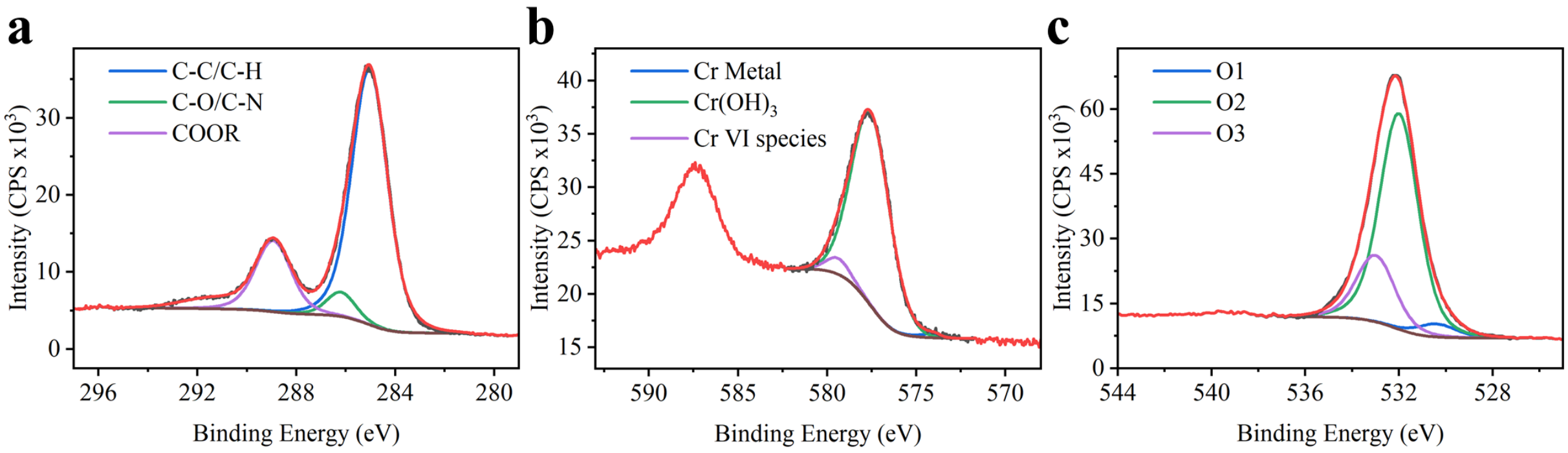

Figure S7. XPS spectra of MIL-101(Cr): (a) C 1s peaks, (b) Cr 2p peaks, and (c) O 1s peaks.

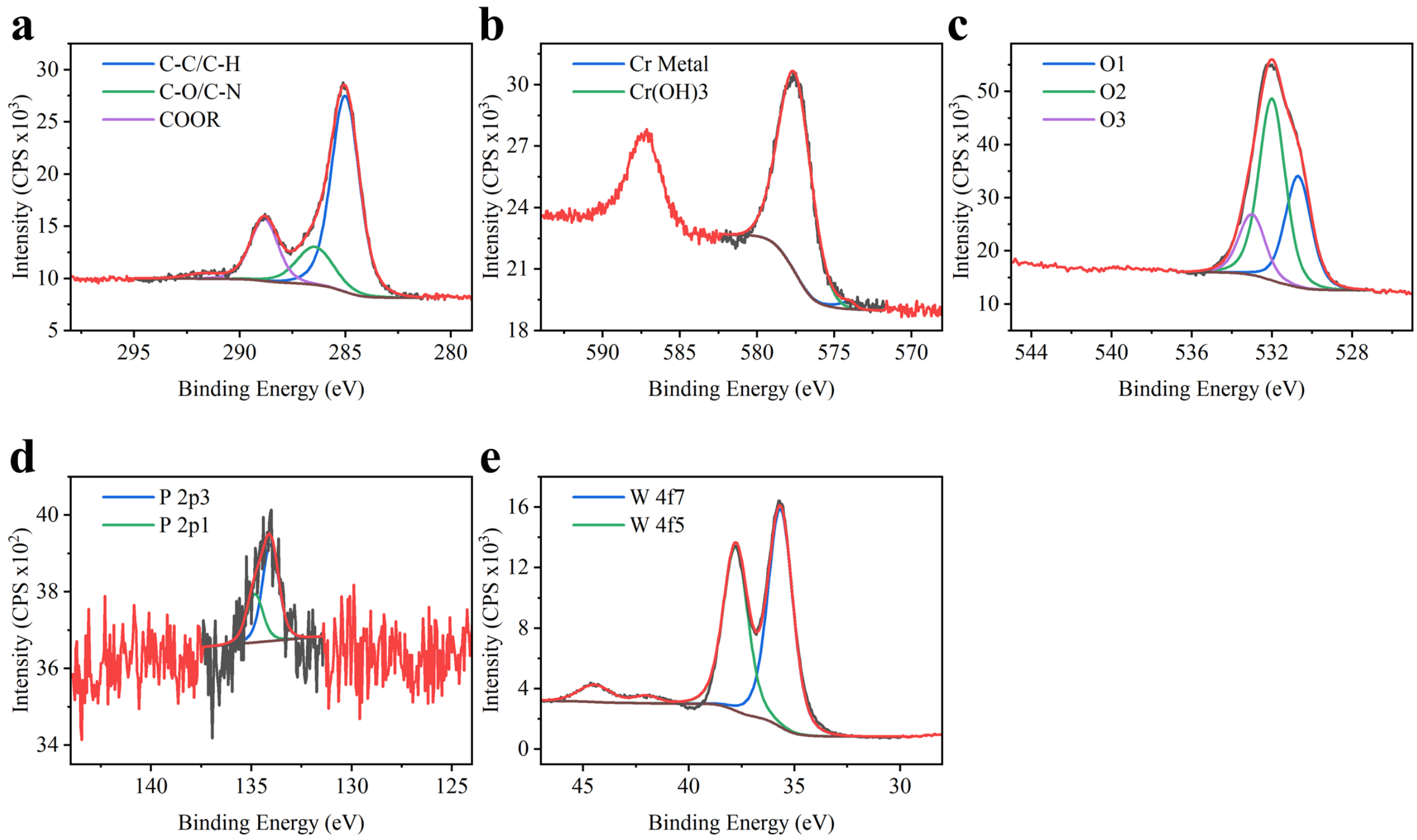

Figure S8. XPS spectra of PW_{12}@MIL-101(Cr): (a) C 1s peaks, (b) Cr 2p peaks, (c) O 1s peaks, (d) P 2p peaks, and (e) W 4d peaks.

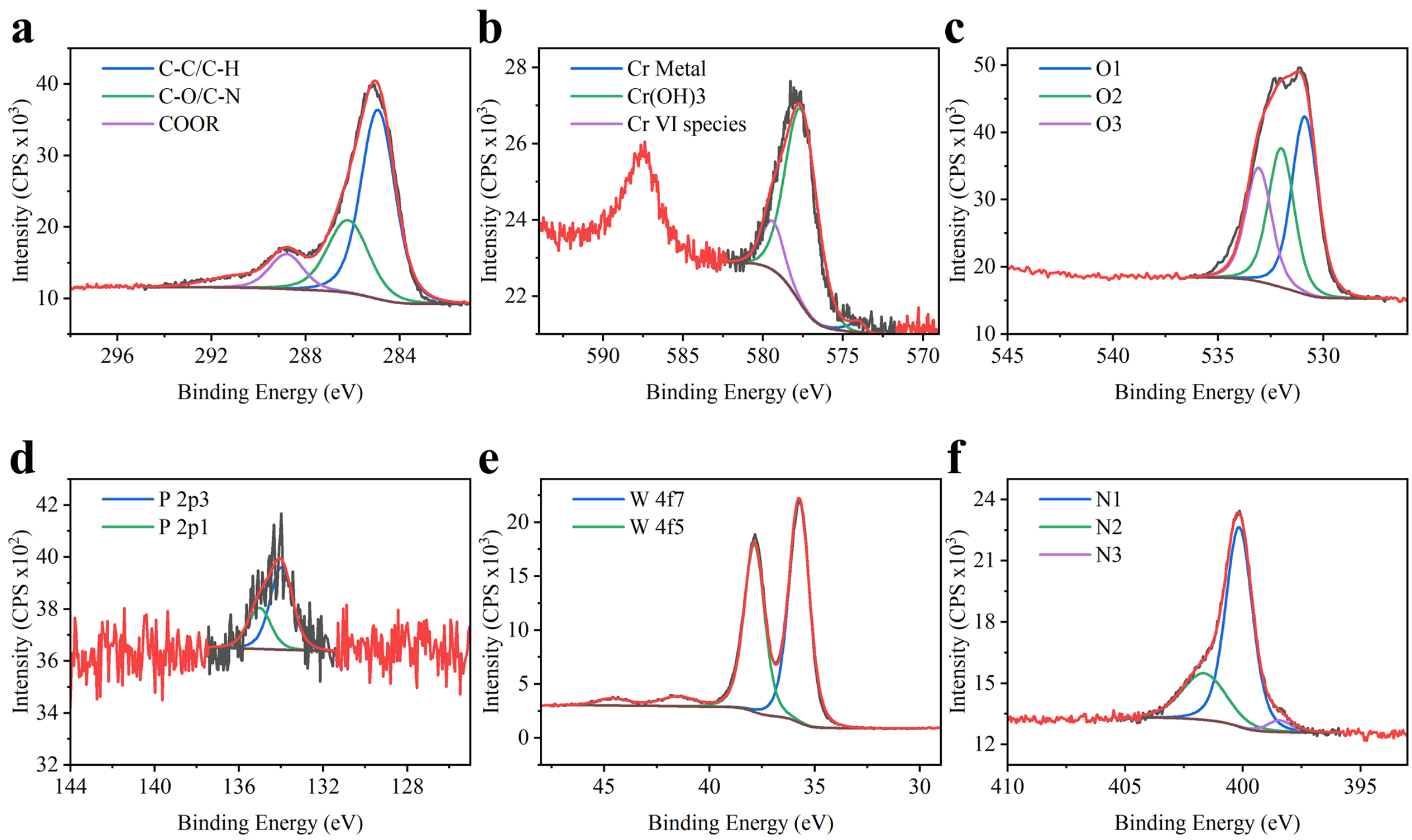

Figure S9. XPS spectra of PW_{12}@MIL-101(Cr)/PPy-MIP: (a) C 1s peaks, (b) Cr 2p peaks, (c) O 1s peaks, (d) P 2p peaks, (e) W 4d peaks, and (f) N 1s peaks.

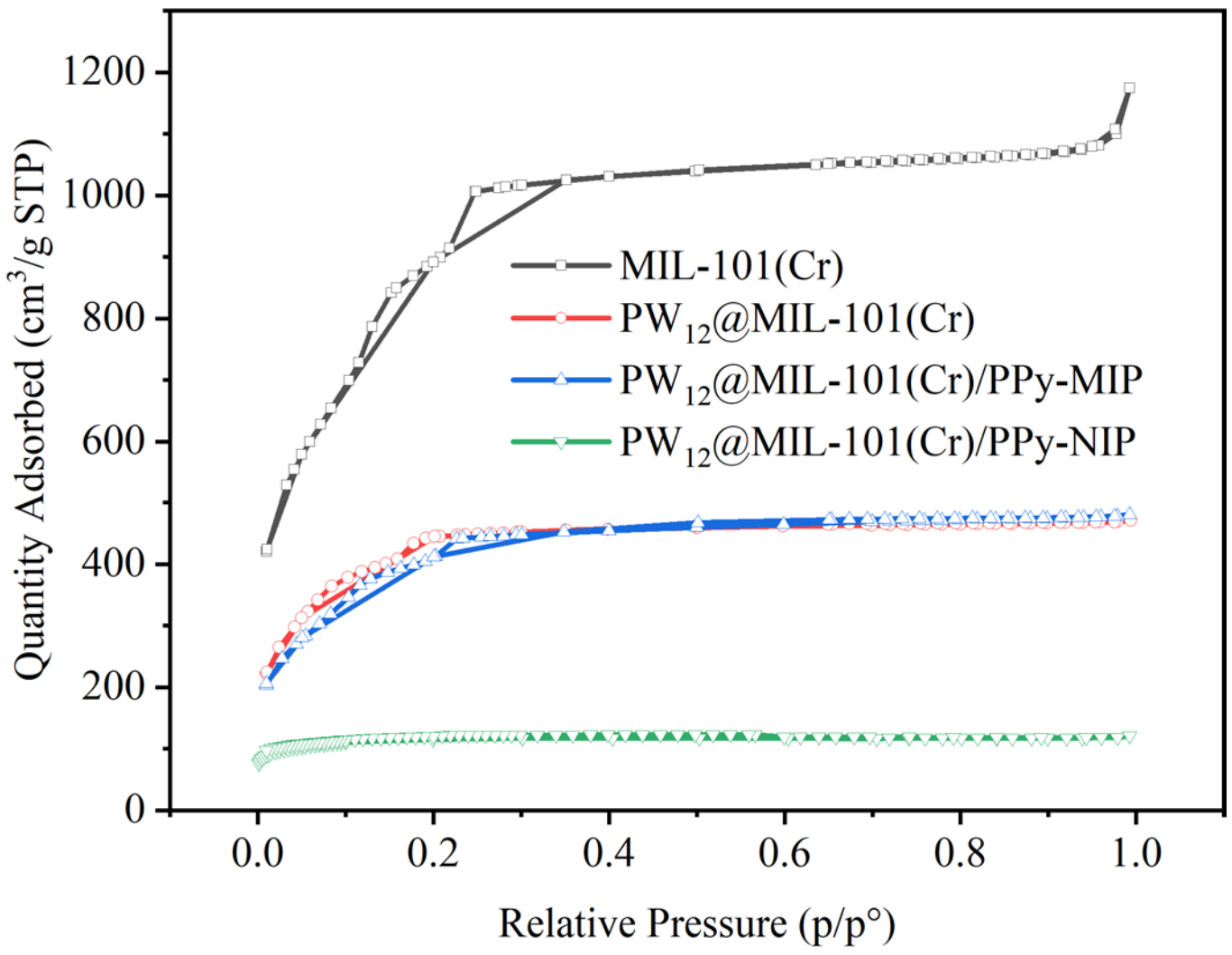

Figure S10. N_2 adsorption/desorption isotherms of PW_{12}@MIL-101(Cr)/PPy-MIP.

Table S1. BET surface area value and fitting parameters for PW_{12}@MIL-101(Cr)/PPy materials.

Materials	BET surface area (m^2 g^{-1})	Pore volume (cm^3 g^{-1})	C constant	Correlation coefficient
MIL-101(Cr)	3223.59 ± 61.68	1.82	48.8416	0.9994
PW_{12}@MIL-101(Cr)	1695.63 ± 23.03	0.73	61.4826	0.9997
PW_{12}@MIL-101(Cr)/PPy MIP	1748.62 ± 33.50	0.74	30.3415	0.9994
PW_{12}@MIL-101(Cr)/PPy NIP	443.79 ± 0.20	0.19	469.7943	0.9999

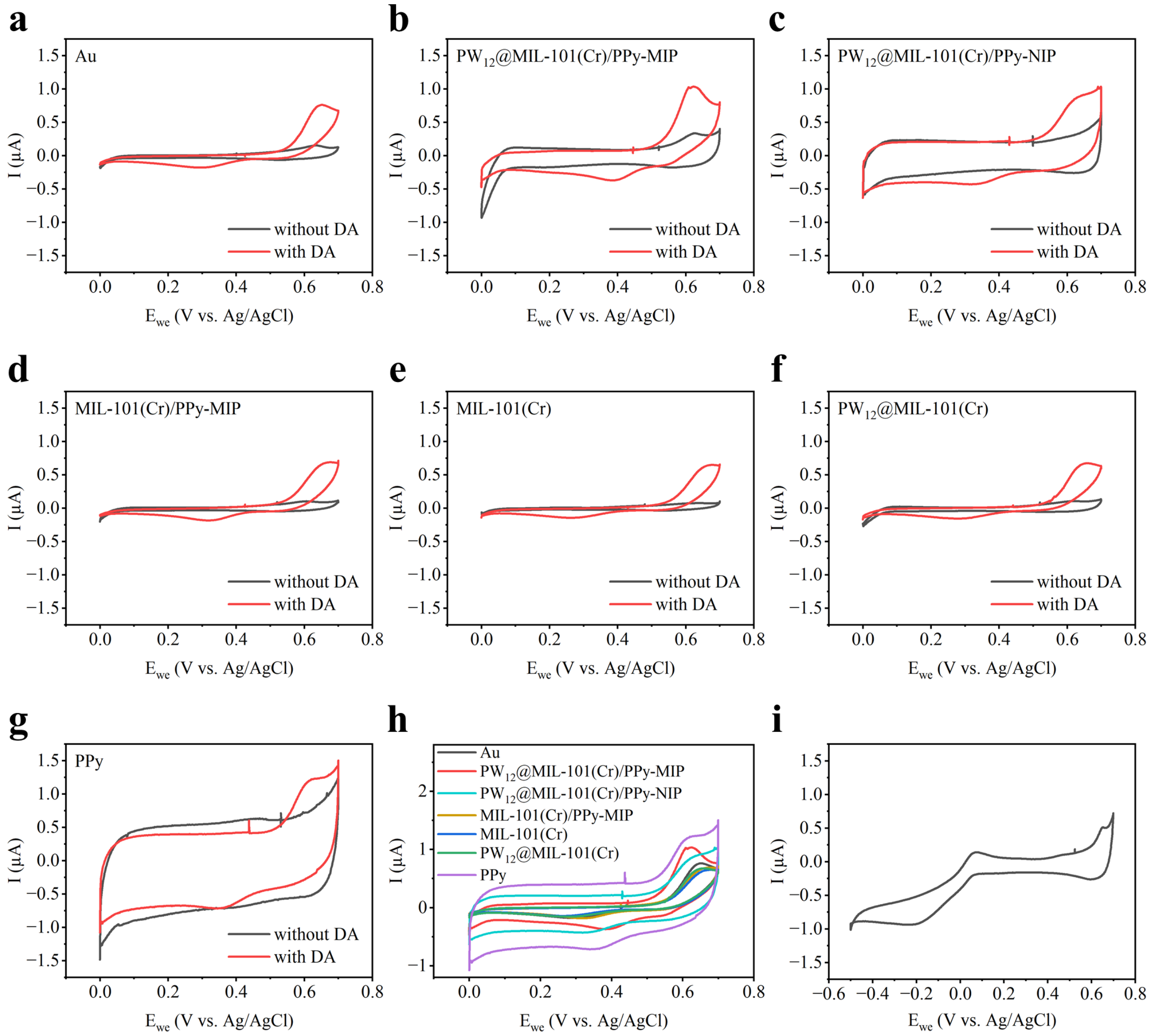

Figure S11. CVs of the (a) Au, (b) PW_{12}@MIL-101(Cr)/PPy-MIP, (c) PW_{12}@MIL-101(Cr)/PPy-NIP, (d) MIL-101(Cr)/PPy-MIP, (e) MIL-101(Cr), (f) PW_{12}@MIL-101(Cr), and (g) PPy WE in 1 M KCl (pH 1.0) in the absence and presence of 200 µM DA at the scan rate of 50 mV s^{-1}. (h) Comparison of the CV responses of various modified WEs in 1 M KCl (pH 1.0) containing 200 µM DA at the scan rate of 50 mV s^{-1}. (i) CV of PW_{12}@MIL-101(Cr)/PPy-MIP electrode recorded in 1 M KCl (without DA) over a wider potential range, showing the redox signal of PW_{12}.

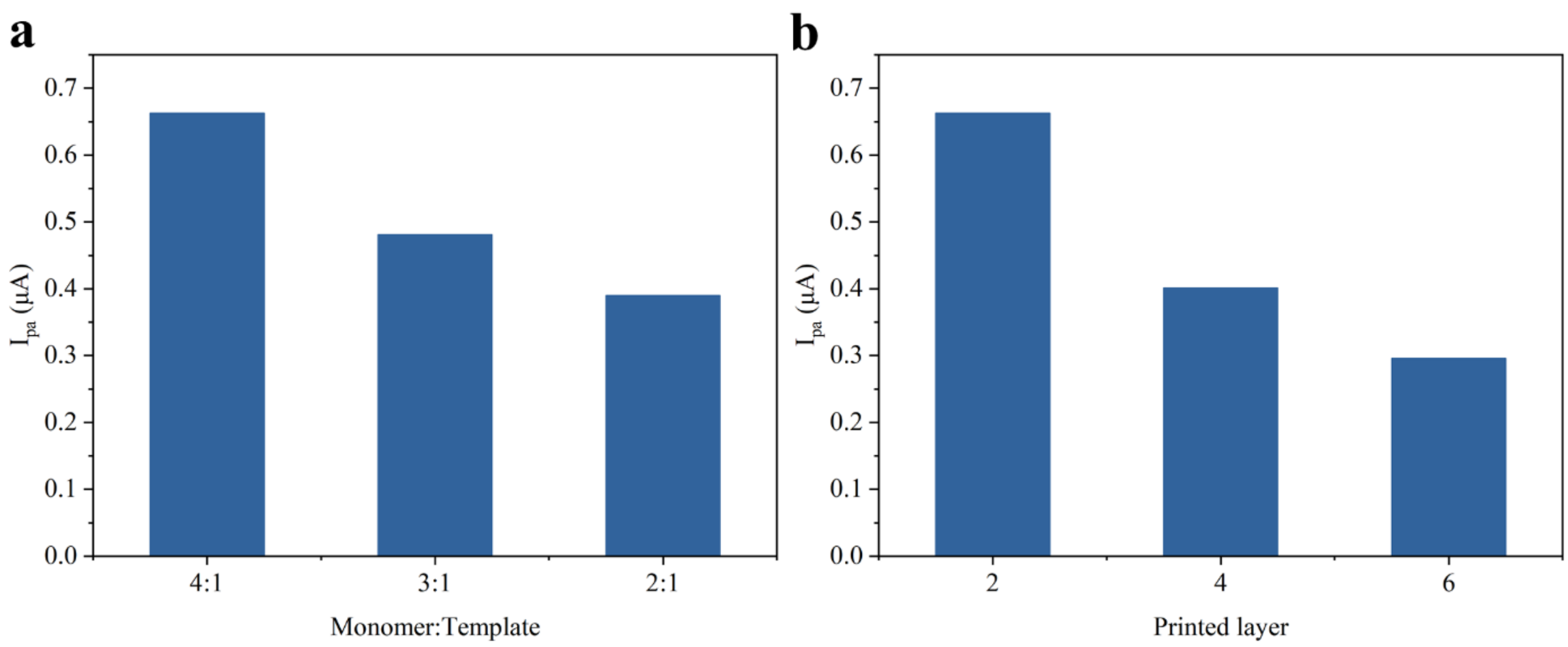

Figure S12. Effects of (a) the monomer-to-template mole ratio in polymer network and (b) the number of PW_{12}@MIL-101(Cr)/PPy-MIP layers on the I_{pc} of the electrochemical response toward 200 μM DA in 1 M KCl (pH 1.0) at the scan rate of 50 mV s^{-1}.

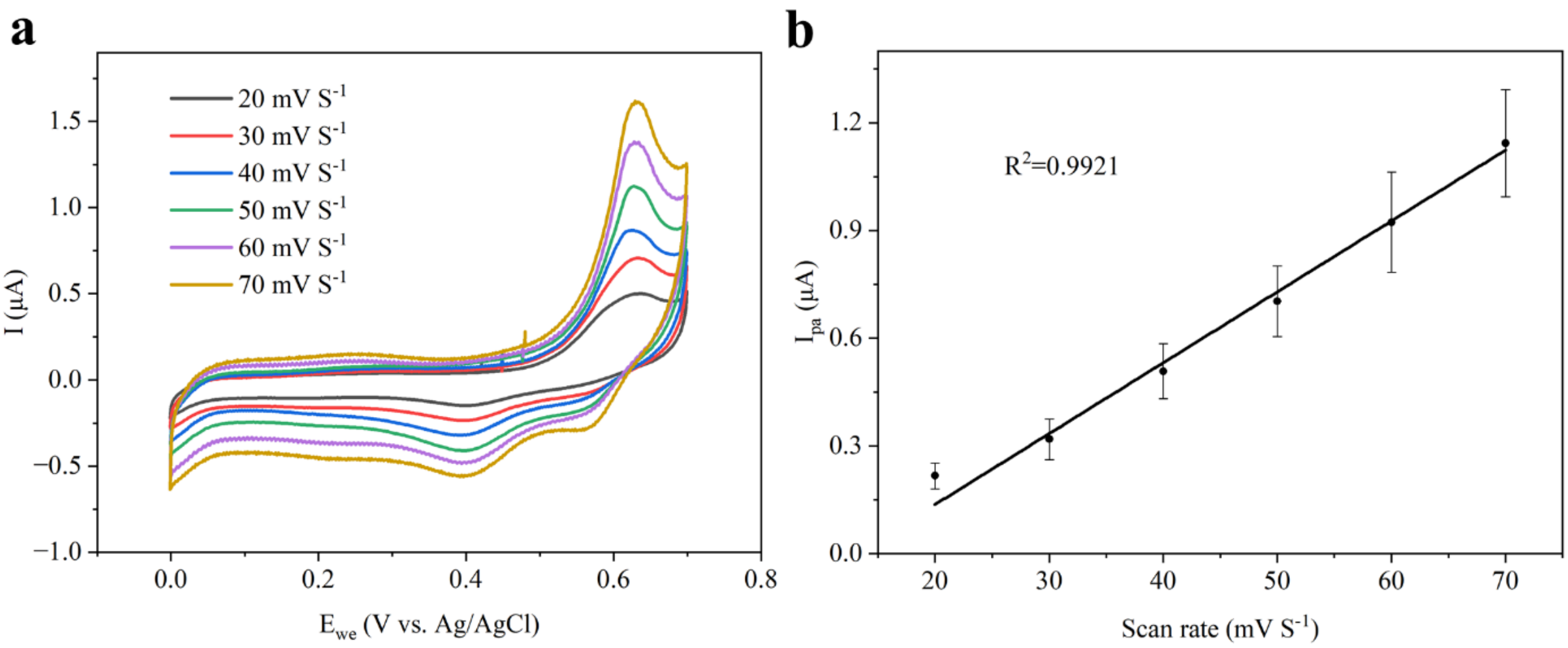

Figure S13. (a) CV curves of the PW_{12}@MIL-101(Cr)/PPy-MIP electrochemical sensor in 1 M KCl (pH 1.0) with 200 μM DA at various scan rates (20-70 mV s^{-1}). (b) Corresponding calibration plot of I_{pa} vs. scan rate for (a).

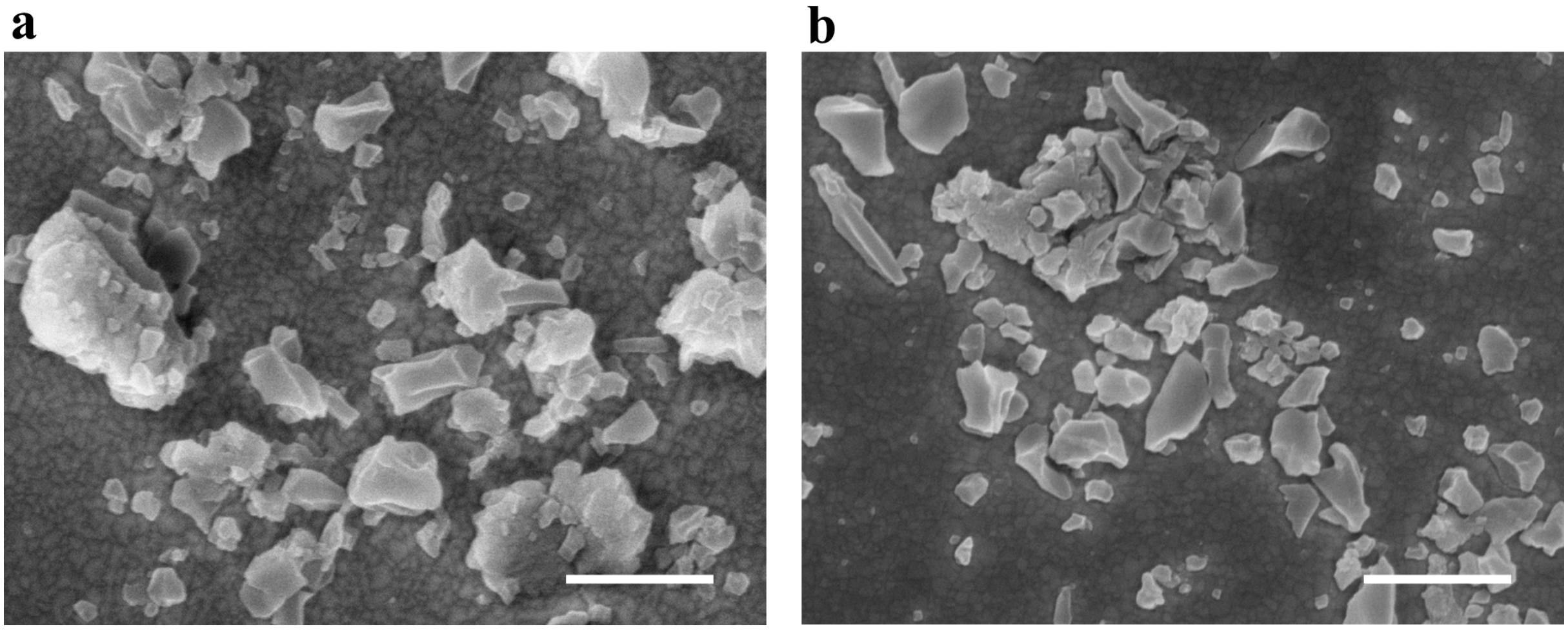

Figure S14. Morphology of PW_{12}@MIL-101(Cr)/PPy-MIP on the WE after (a) 50 consecutive scan cycles and (b) six regeneration cycles. Scale bar: 1 μm.

Table S2. Comparison of sensing performances of different composite-modified DA sensors

Materials	Electrode	Electrode diameter (mm)	Functionalization method	LOD (μM)	Selectivity	Stability	Regeneration ability/ Reusebility	Reproducibility	Ref.
HKUST-1	Glassy carbon electrode (GCE)	–	Drop casting	0.15	AA	2 months	–	Yes	[1]
CuNi-MOF@rGO	Polyimide electrode	–	Drop casting	9.41	UA, Glu, tyrosine, glycine, urea	7 days	–	Yes	[2]
Au@Cu-MOF	Screen-printed carbon electrode (SPCE)	–	Drop casting	3.4	Glu, AA, UA, fructose, urea, sucrose	30 days	Yes	Yes	[3]
PPy/ZIF-67-MIPs/Nafion	GCE	–	Drop casting	0.0308	AA, UA, vitamin B6, oxytetracycline, folic	2 months	Yes	Yes	[4]

					acid, catechin				
$V_{10}O_{28}$@NU-902	Fluorine doped tin oxide electrode	5×5	Drop casting	2.1	–	20 cycles	–	–	[5]
$\{[Mo_5P_2O_{23}][Cu(phen)(H_2O)]_3 \cdot 5H_2O\}$/rGO	GCE	–	Drop casting	0.0804	AA, UA, Glu	100 cycles	–	Yes	[6]
$(P_2W_{16}V_2$-AuPd/PEI$)_n$	ITO electrode	–	Layer-by-layer self-assembly	0.217	AA, UA, Glu, L-cysteine	300 cycles	Yes	Yes	[7]
rGO/PPy	GCE	3	Drop casting	0.61	–	–	–	–	[8]
PVP/ZnO	SPCE	–	Electrodeposition	1.3375	AA, UA	15 days	Yes	Yes	[9]
TiO_2	Screen-printed Ti electrode	4	Electrodeposition	20	Glu, AA, UA	–	–	–	[10]
GONRs + PEDOT:PSS	SPCE	2.5	Syringe printing	0.03	AA, UA	–	Yes	Yes	[11]
PEDOT	Laser scribed graphene electrode	2	Electrodeposition	15	AA, UA	3 days	Yes	Yes	[12]

PW_{12}@MIL-101(Cr)/PPy-MIP	Laser-ablated Au electrode	1	Capillary printing	59.03	AA, UA	50 cycles	Yes	Yes	This work

Table S3. I_{pa} and imprinting factor values of PW_{12}@MIL-101(Cr)/PPy-MIP/NIP sensors for DA detection

Analyte	I_{pa} (μA)		Imprinting factor
	PW_{12}@MIL-101(Cr)/PPy-MIP	PW_{12}@MIL-101(Cr)/PPy-NIP	
DA	0.662803	0.258656	2.562488

Reference

[1] J. Li, J. Xia, F. Zhang, Z. Wang, Q. Liu, “A Novel Electrochemical Sensor Based on Copper-based Metal-Organic Framework for the Determination of Dopamine” J. Chin. Chem. Soc. 2018, 65, 743–749.

[2] C. Wang, Y. Zhang, Y. Liu, X. Zeng, C. Jin, D. Huo, J. Hou, C. Hou, “A wearable flexible electrochemical biosensor with CuNi-MOF@rGO modification for simultaneous detection of uric acid and dopamine in sweat” Anal. Chim. Acta 2024, 1299, 342441.

[3] F. Zhou, H. N. Lim, I. Ibrahim, N. A. Endot, E. A. Malek, N. S. K. Gowthaman, “Simultaneous Electrochemical Detection of Dopamine and Uric Acid via Au@Cu-Metal Organic Framework” ChemPlusChem 2024, 89, e202300686.

[4] W. Zhang, D. Duan, S. Liu, Y. Zhang, L. Leng, X. Li, N. Chen, Y. Zhang, “Metal–Organic Framework-Based Molecularly Imprinted Polymer as a High Sensitive and Selective Hybrid for the Determination of Dopamine in Injections and Human Serum Samples” Biosens. Bioelectron. 2018, 118, 129–136.

[5] W. H. Ho, T.-Y. Chen, K. Otake, Y.-C. Chen, Y.-S. Wang, J.-H. Li, H.-Y. Chen, C.-W. Kung, “Polyoxometalate Adsorbed in a Metal–Organic Framework for Electrocatalytic Dopamine Oxidation” Chem. Commun. 2020, 56, 11763–11766.

[6] W. Zhang, G. Jia, Z. Li, C. Yuan, Y. Bai, D. Fu, “Selective Electrochemical Detection of Dopamine on Polyoxometalate-Based Metal–Organic Framework and Its Composite with Reduced Graphene Oxide” Adv. Mater. Interfaces 2017, 4, 1601241.

[7] C. Zhou, S. Li, W. Zhu, H. Pang, H. Ma, “A Sensor of a Polyoxometalate and Au–Pd Alloy for Simultaneously Detection of Dopamine and Ascorbic Acid” Electrochimica Acta 2013, 113, 454–463.

[8] M. Akin, R. Bayat, M. Bekmezci, Z. K. Coguplugil, F. Sen, M. Baghayeri, A. Kaffash, F. Tehranejad-Javazmi, I. Sheikhshoaie, “The use of polymer/carbon based material as an efficient and low-cost electrochemical sensor for rapid electrochemical detection of dopamine” Carbon Lett. 2023, 33, 1993–2001.

[9] B. S. Devi, R. Karthikeyan, M. Anitha, S. Prakash, “Enhanced electrochemical sensing of dopamine using PVP/Green synthesized ZnO Nanocomposite-Modified Screen-Printed carbon Electrode: A promising tool for Neurochemical analysis” Mater. Sci. Eng. B 2025, 317, 118219.

[10] F. Tavella, C. Ampelli, S. G. Leonardi, G. Neri, "Photo-Electrochemical Sensing of Dopamine by a Novel Porous TiO2 Array-Modified Screen-Printed Ti Electrode" Sensors 2018, 18, 3566.

[11] C.-H. Su, C.-L. Sun, Y.-C. Liao, "Printed Combinatorial Sensors for Simultaneous Detection of Ascorbic Acid, Uric Acid, Dopamine, and Nitrite" ACS Omega 2017, 2, 4245–4252.

[12] G. Xu, Z. A. Jarjes, V. Desprez, P. A. Kilmartin, J. Travas-Sejdic, "Sensitive, selective, disposable electrochemical dopamine sensor based on PEDOT-modified laser scribed graphene" Biosens. Bioelectron. 2018, 107, 184–191.
